# A patient stratification signature mirrors the immunogenic potential of high grade serous ovarian cancers

**DOI:** 10.1186/s12967-024-05846-9

**Published:** 2024-11-20

**Authors:** Laurel K. Berry, Ashok K. Pullikuth, Kristen L. Stearns, Yuezhu Wang, Calvin J. Wagner, Jeff W. Chou, Janelle P. Darby, Michael G. Kelly, Raghvendra Mall, Ming Leung, Julia Chifman, Lance D. Miller

**Affiliations:** 1 Department of Obstetrics and Gynecology, Section on Gynecologic Oncology, Wake Forest University School of Medicine, Winston-Salem, NC 27157 USA; 2 Department of Cancer Biology, Wake Forest University School of Medicine, Winston-Salem, NC 27157 USA; 3 Atrium Health Wake Forest Baptist Comprehensive Cancer Center, Winston-Salem, NC 27157 USA; 4 Department of Immunology, St. Jude Children’s Research Hospital, Memphis, TN 38105 USA; 5 Present Address: Biotechnology Research Center, Technology Innovation Institute, P.O. Box 9639, Abu Dhabi, United Arab Emirates; 6 Department of Mathematics and Statistics, American University, Washington, DC 20016 USA

**Keywords:** High grade serous ovarian cancer, Immunogenicity, Immune activation, Immunosuppression, Immune evasion, Mutational burden, Patient stratification, Gene signature

## Abstract

**Background:**

While high-grade serous ovarian cancer (HGSC) has proven largely resistant to immunotherapy, sporadic incidents of partial and complete response have been observed in clinical trials and case reports. These observations suggest that a molecular basis for effective immunity may exist within a subpopulation of HGSC. Herein, we developed an algorithm, CONSTRU (Computing Prognostic Marker Dependencies by Successive Testing of Gene-Stratified Subgroups), to facilitate the discovery and characterization of molecular backgrounds of HGSC that confer resistance or susceptibility to protective anti-tumor immunity.

**Methods:**

We used CONSTRU to identify genes from tumor expression profiles that influence the prognostic power of an established immune cytolytic activity signature (CYTscore). From the identified genes, we developed a stratification signature (STRATsig) that partitioned patient populations into tertiles that varied markedly by CYTscore prognostic power. The tertile groups were then analyzed for distinguishing biological, clinical and immunological properties using integrative bioinformatics approaches.

**Results:**

Patient survival and molecular measures of immune suppression, evasion and dysfunction varied significantly across STRATsig tertiles in validation cohorts. Tumors comprising STRATsig tertile 1 (S-T1) showed no immune-survival benefit and displayed a hyper-immune suppressed state marked by activation of TGF-β, Wnt/β-catenin and adenosine-mediated immunosuppressive pathways, with concurrent T cell dysfunction, reduced potential for antigen presentation, and enrichment of cancer-associated fibroblasts. By contrast, S-T3 tumors exhibited diminished immunosuppressive signaling, heightened antigen presentation machinery, lowered T cell dysfunction, and a significant CYTscore-survival benefit that correlated with mutational burden in a manner consistent with anti-tumor immunoediting. These tumors also showed elevated activity of DNA damage/repair, cell cycle/proliferation and oxidative phosphorylation, and displayed greater proportions of Th1 CD4 + T cells. In these patients, but not those of S-T1 or S-T2, validated predictors of immunotherapy response were prognostic of longer patient survival. Further analyses showed that STRATsig tertile properties were not explained by known HGSC molecular or clinical subtypes or singular immune mechanisms.

**Conclusions:**

STRATsig is a composite of parallel immunoregulatory pathways that mirrors tumor immunogenic potential. Approximately one-third of HGSC cases classify as S-T3 and display a hypo-immunosuppressed and antigenic molecular composition that favors immunologic tumor control. These patients may show heightened responsiveness to current immunotherapies.

**Supplementary Information:**

The online version contains supplementary material available at 10.1186/s12967-024-05846-9.

## Background

Ovarian cancer is the leading cause of gynecologic cancer-related death in developed countries and the 5th leading cause of cancer death among women in the US [1, 2]. The most common subtype of epithelial ovarian cancer (EOC) is high grade serous ovarian cancer (HGSC) which accounts for three quarters of all ovarian cancer cases and is largely responsible for the high rates of cancer-related deaths [3]. The vast majority of HGSC patients present with Stage III or IV disease. A combination of aggressive surgery and platinum-based chemotherapy has remained the standard of care first-line treatment over the last three decades, yet these strategies are associated with high rates of recurrence and low rates of long-term survival. Recurrent disease after surgery and chemotherapy is expected and incurable for 75% of patients, leading to a five-year survival of approximately 30% for patients with advanced stage disease at diagnosis [4]. There remains a critical need for more effective treatments in both the upfront and recurrent settings.

Immunotherapy is an attractive treatment which has had success in other solid tumor disease sites such as skin, lung, endometrial, breast, colorectal, and urologic cancers [5–9]. There has been interest in the use of immune checkpoint inhibitors (ICI) in HGSC, as the levels of tumor infiltrating lymphocytes (TILs) and specific TIL subsets have been significantly correlated with survival [10]. Unfortunately, HGSC response to ICI has, thus far, been mostly ineffective with overall response rates (ORR) of 8–28% [11, 12]. However, it has been reported that HGSC patients who initially respond to ICI experience durable responses of greater than 6 months [13], and multiple case reports have described remarkable, durable responses to single agent immunotherapy [14, 15]. These observations suggest that some fraction of HGSC cases may possess inherent molecular attributes capable of eliciting protective anti-tumor immunity.

Biomarkers such as tumor mutational burden (TMB), PD-L1 expression, deficiency in mismatch repair (dMMR), and the presence of high microsatellite instability (MSI-H) have emerged as clinically relevant predictors of immunotherapy efficacy [16]. HGSC, however, does not have a particularly high TMB [17], rarely presents as dMMR or MSI-H [18], and PD-L1, even when elevated, has not proven to be a reliable predictor of ICI response in HGSC [11]. Thus, HGSC immunogenicity may depend largely on immune activating and suppressing signals that we do not fully understand [19].

TIL abundance and activity, as determined by tumor histopathological assessment, has been associated with immune-mediated survival in many solid tumor types [20–22], including HGSC [23]. Recent reports by us and others [24–27] have leveraged large-scale tumor gene expression profiling studies to identify immune gene signatures that quantify the relative abundance and functional orientation of distinct TIL populations. A number of these signatures, and particularly those that reflect TIL biology, have demonstrated robust associations with both patient survival and immunotherapy response in a variety of cancer types, sparking interest in their use as clinical biomarkers of ICI efficacy [28–30]. Recently, a quantitative gene-based measure of immune cytolytic activity was devised for studying relationships between anti-tumor immunity, gene mutations and mechanisms of tumor resistance [31]. Known as the CYT gene signature (CYTscore), it is based on the transcript levels of two key cytolytic effectors, Granzyme A (GZMA) and Perforin 1 (PRF1), which are both dramatically upregulated upon effector cell activation. In a series of reports on the prognostic power of the CYTscore, significant associations with patient overall and disease-free survival were observed in multiple cancer types [32–36], indicative of its utility as a biomarker of protective anti-tumor immunity.

Recently, we and others have endeavored to better understand the effects of tumor-patient heterogeneity on biomarker performance. In this study, we investigate the applicability of a new biomarker discovery strategy that seeks to uncover molecularly distinct patient subgroups inherent to a population that, for a given biomarker, can be separated out on the basis of biomarker performance. Using this strategy, we uncover heterogeneous states of immunoregulation in HGSC that differ by potential for immunologic tumor control.

## Methods

### Expression data acquisition, processing and annotation

Six curated data sets of tumor expression profiles and corresponding clinical data from high-grade serous ovarian cancer (HGSC) patients were analyzed. Protocols for patient consent and sample acquisition were approved by Institutional Review Boards at each site. The OV1 data set comprises 431 HGSC cases profiled on the Affymetrix U133A platform as part of the early TCGA initiative [37] and accessible via Gene Expression Omnibus (GEO) accession number GSE82191. The 431 cases represent the subset of 527 cases annotated as high-grade (grade 2 or 3) serous histology with > 3 months follow-up, and not identified as redacted in Table S1 of the TCGA clinical update report [38]. Data was normalized by the RMA method [39] as implemented in the R package *affy* [40] provided by Bioconductor [41].

OV2 consists of 227 HGSC cases associated with the Australian Ovarian Cancer Study and profiled on the Affymetrix U133 Plus 2.0 platform [42] with GEO accession GSE9891. The 227 cases represent the subset of 285 cases annotated as high-grade serous histology with accompanying overall survival data and > 3 months follow-up. Data was normalized by RMA.

OV3 is a batch-corrected compilation of three smaller data sets (GSE3149 [43], n = 110; GSE14764 [44], n = 66; and GSE30161 [45], n = 45) profiled on the Affymetrix U133A [43, 44] or U133 PLUS 2.0 [45] platforms, and consists of 221 HGSC cases in total. The 221 cases are the subset of 255 cases annotated as high-grade serous histology with accompanying overall survival data. Data sets were normalized by RMA [43, 45] or MAS5.0 [44]. Original CEL files for GSE3149 [43] were accessed at https://bioinformatics.mdanderson.org/Supplements/ReproRsch-Ovary/Modified/DressmanArchive/index.html and corrected for run date batch effects as recommended by Baggerly and colleagues [46]. The ComBat empirical Bayes method [47] was used to correct for batch effects. Updated corresponding clinical data were obtained via curatedOvarianData v3.18 (https://www.bioconductor.org/packages/release/data/experiment/ html/curatedOvarianData.html) [48].

OV4 consists of 174 HGSC cases profiled on the Agilent-014850 Whole Human Genome Microarray 4 × 44 K G4112F platform [49] with GEO accession GSE53963. Data were normalized by Linear/LOWESS normalization as reported [49].

The OV5 data set comprises 260 HGSC cases associated with the Japanese Serous Ovarian Cancer Study Group [50] and profiled on the Agilent-014850 Whole Human Genome Microarray 4 × 44 K G4112F platform with GEO accession GSE32062. Data were normalized by the scaling method as published [50].

OV6 consists of 212 HGSC cases associated with the ICON7 (International Collaboration on Ovarian Neoplasms) multicenter clinical trial [50] and profiled on the Illumina HumanHT-12 WG-DASL V4.0 R2 expression beadchip [51] with GEO accession GSE140082. Data were normalized by quantile normalization as reported [51]. Patient survival data and/or other clinical annotations for OV1, OV2, OV3 and OV5 were provided by the Bioconductor curatedOvariandata package [48]. Updated survival data for OV1 was used as published in Table S1 of the TCGA clinical update report [38]. For OV4 and OV6, patient survival data and other clinical annotations were obtained at GSE53963 and GSE140082, respectively.

The OTTA (Ovarian Tumor Tissue Analysis) consortium data set is derived from an international multi-site HGSC cohort consisting of 3,769 tumor specimens annotated for survival and other clinical characteristics [52, 53]. The tumor specimens were profiled on the NanoString n-Counter platform for the expression of 513 genes, and the normalized gene expression profiles were retrieved from GEO accession GSE132342. Details regarding NanoString data quality assurance, monitoring for batch effects, single-patient normalization by reference genes and reference pools, and metrics for sample inclusion are described by Talhouk and colleagues [53].

### Derivation of gene signatures

Gene identities corresponding to array probes or probe sets were standardized as follows. The Ensembl BioMart (Ensembl Genes 102 DATABASE, Human genes (GRCh38.p14) DATASET) [54] was used to annotate all human genes with Hugo Gene Nomenclature Committee (HGNC) approved gene names and symbols, as well as corresponding probe or probe set IDs for the Affymetrix, Agilent, and Illumina array platforms. Gene name and symbol updates were performed using the HGNC Multi-symbol checker tool (https://www.genenames.org/) [55]. Gene signature scores were obtained by computing the mean of the log2 expression values of the genes comprising a gene signature. The cytolytic activity signature (CYTscore) was defined as the mean of PRF1 (214617_at) and GZMA (205488_at) as described by Rooney and colleagues [31].

The APM gene signature predictive of ICI response in non-small cell lung cancer and melanoma [56] was defined as the sum of the log2 z-scores of the 8 genes: B2M, CALR, NLRC5, PSMB9, PSME1, PSME3, RFX5 and HSP90AB1. T cell dysfunction scores were computed as described by Jiang [30] and Fu [57] using the Tumor Immune Dysfunction and Exclusion (TIDE) web platform (http://tide.dfci.harvard.edu/) [58].

### The CONSTRU algorithm and STRATsig candidate gene ranking by parity score

**Fig. 1:**
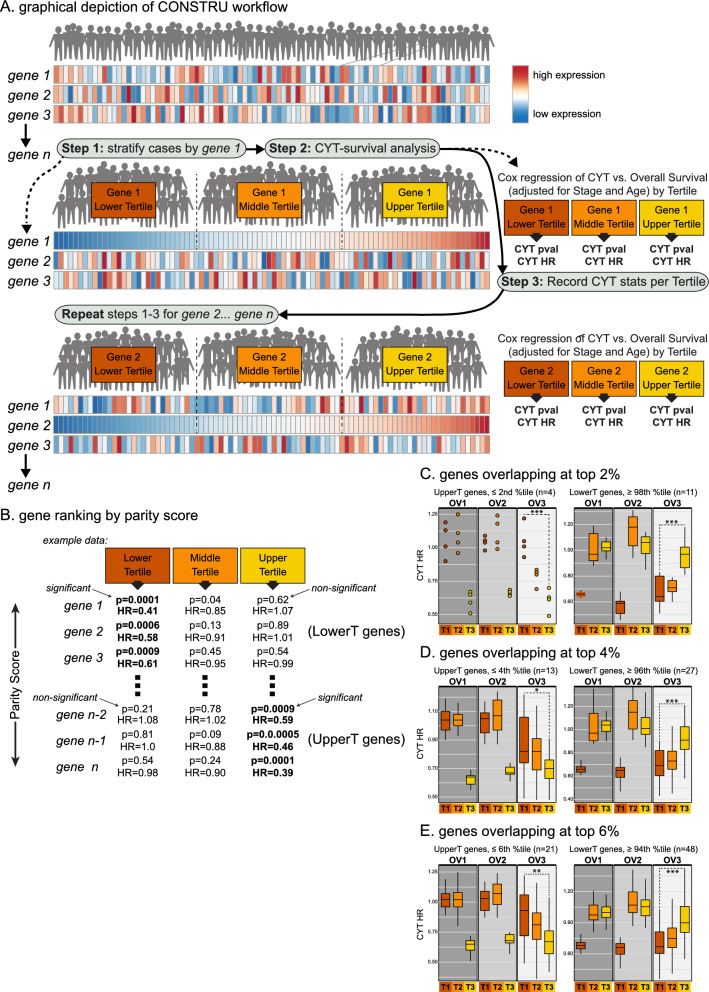
Description of CONSTRU workflow and strategies for gene ranking and selection. **A** Graphical depiction of CONSTRU workflow illustrating the iterative process of ranking cases into gene expression tertiles, then computing CYTscore-survival statistics for each tertile of each gene. **B** Illustration of gene ranking by parity score and partitioning of LowerT and UpperT genes. **C**–**E** Assessing reproducibility of top UpperT and LowerT genes. For each gene selected by parity score percentile rank thresholds (2%, 4%, and 6%), and overlapping between OV1 and OV2, the CYTscore hazard ratios (HR) are plotted for each gene’s tertile, in each data set. The OV3 data set was used to assess the general reproducibility of the gene tertile-specific CYTscore-survival associations observed in OV1 and OV2. *P < 0.05 and > 0.01; **P < 0.01 and  > 0.001; ***P < 0.001

CONSTRU (Computing Prognostic Marker Dependencies by Successive Testing of Gene-Stratified Subgroups) is an algorithm designed for use with tumor gene expression profiling data, to empirically discover groups of tumors designated by discrete gene expression-based partitions for which a continuous or categorical prognostic variable will or will not significantly associate with patient survival (see Fig. 1). The algorithm takes as input a tumor-gene expression data matrix with tumors (columns) annotated for survival time and event and available prognostic variables. The expression data for each gene (rows) is used to organize tumors into groups based on the gene’s relative expression level. In the current implementation, tumors are grouped according to gene expression tertiles, thereby representing a standardized measure of low, intermediate or high expression. For each gene tertile, a multivariable Cox proportional hazards regression model is fitted to the data, and the significance (p-value) and directionality (hazard ratio) of the association between the CYTscore (continuous; mean expression of GZMA and PRF1) and patient OS is computed with adjustment for other prognostic factors, including patient age (continuous), FIGO stage (low (stage I or II), high (stage III or IV), NA), and surgical debulking status (optimal, suboptimal, NA).

Once the Cox model statistics for CYTscore are computed for each tertile of each gene, the algorithm outputs a text file displaying the tertile-specific statistics for all genes. This file is then used to rank genes according to their tertile-specific Cox statistics using a parity score developed to distinguish favorable outcome-associated genes within tertiles, with an emphasis on the largest difference between lower (T1) or upper (T3) tertiles with respect to CYTscore prognostic power. The parity score uses CYTscore-survival Cox p-values and hazard ratios according to the following formula: *(− log*_*10*_*P/HR)*_*[T1]*_* – (− log*_*10*_*P/HR)*_*[T3]*_ to generate a combined measure of significance (-log_10_P) and effect size (HR, hazard ratio) for each tertile (T1, T3) and calculates the delta between them. The larger this delta, the greater the difference between T1 and T3 with respect to CYTscore significance and effect size. Higher parity scores equate with genes that have greater CYTscore-survival associations in T1 (termed LowerT genes), while lower parity scores reflect genes with greater CYTscore-survival associations in T3 (termed UpperT genes). The parity scores (see also alternate parity score formulas at the GitHub link) are then assigned percentile ranks as a function of all genes used in the analysis, thus allowing a standardized approach for comparing genes across data sets. In parallel, for comparative purposes, the algorithm also considers CYTscore as a continuous variable for computing the Cox proportional hazards interaction term and significance for each gene in the data set. The R code for the CONSTRU algorithm can be found at GitHub (https://github.com/chifman/Constru).

As the goal is to stratify patients based on relationships between gene expression levels and the prognostic significance of a gene signature, genes having expression patterns correlated with the gene signature are poor candidates, as the tumor groups defined by the resulting gene tertiles will each exhibit a compressed distribution of gene signature values. For example, if a gene is positively correlated with CYTscore, then the gene’s lower tertile will be enriched for tumors with low CYTscore values, while the gene’s upper tertile will be enrich for tumors with high CYTscore values. Therefore, after running CONSTRU, genes were filtered based on their inherent positive or negative correlations with CYTscore. We used an empirical threshold of Pearson’s correlation coefficient of > 0.15 or < -0.15 to exclude genes from analysis. When applied to the OV1 and OV2 data sets, a common set of 8,048 probe sets corresponding to 7,866 genes remained for parity score percentile rank comparisons between OV1 and OV2. The OV1 and OV2 data sets were selected for use in the training process for the following reasons: 1) they share a large common set of gene probes with identical sequence (i.e., 22,277 probe sets), 2) patient overall survival rates are not significantly different between OV1 and OV2, and 3) in terms of sample size, they are among the largest HGSC whole-genome expression profiling data sets published to date.

The parity score was used to select UpperT and LowerT genes identified within the 4th-percentile cutoffs of both OV1 and OV2. The UpperT gene signature was defined as the mean of C8orf33 (218187_s_at), CDC42EP4 (218062_x_at), DDX21 (208152_s_at), DNAJC9 (213092_x_at, 213088_s_at), MEGF6 (213942_at), NCAPD3 (212789_at), RAF1 (201244_s_at), RTF1 (212302_at), TPD52 (201691_s_at), TUBGCP4 (211337_s_at), UBP1 (218082_s_at), UQCRB (209066_x_at) and ZNF250 (213858_at). The LowerT gene signature was defined as the mean of ALB (211298_s_at), AMACR (209424_s_at), APBB2 (40148_at), BAG2 (209406_at), BDH2 (218285_s_at), CAMK2N1 (218309_at), CAV2 (203323_at), CDC14B (221556_at), DNAJB4 (203811_s_at), EVA1B (220134_x_at), FAT4 (219427_at), GALC (204417_at), HAS2 (206432_at), HOXA9 (214651_s_at), MFGE8 (210605_s_at), NPTXR (213040_s_at), OSR2 (213568_at), PCGF1 (210023_s_at), PCOLCE2 (219295_s_at), PEPD (202108_at), PID1 (219093_at), PLAGL1 (207943_x_at), POGLUT2 (219479_at), STAM2 (209649_at), TRPC1 (205803_s_at), WDFY3 (212602_at) and WNT7A (210248_at). STRATsig scores were computed by subtracting the LowerT score from the UpperT score. For non-Affymetrix data sets, the probes corresponding to these genes were used to compute STRATsig scores; when a gene was represented by more than one probe, the probe expression values were averaged prior to computing LowerT and UpperT scores. In the case of OV4 and OV5, probes representing 2 of the 27 genes of the LowerT signature, EVA1B and POGLUT2, were not available for inclusion in the STRATsig calculation.

### Pathway enrichment

In each of the six data sets (OV1-OV6), gene expression patterns were assessed for correlation (Pearson) with STRATsig, and the top 2% of genes most positively or negatively correlated with STRATsig were identified. The DAVID (Database for Annotation, Visualization and Integrated Discovery) Knowledgebase v6.8 and gene functional annotation tools [59] were used to uncover significantly enriched pathways and gene ontologies in the 2nd-percentile gene lists (i.e., the positively or negatively correlated gene lists). The integrated training group (OV1, OV2, OV3) and test group (OV4, OV5, OV6) were used to compute activation levels of 54 cancer-related pathways in the context of STRATsig tertiles (T1, T2, T3) and CYTscore tertiles (Lo, Mid, Hi). For this work, single-sample gene set enrichment analysis (ssGSEA) was performed using the GSVA (v1.42.0) R package [60] with the ‘gsva’ function kernel density parameter set as ‘Gaussian’ kernel. Gene sets used to compute pathway activation scores were obtained from multiple sources, including the 24 hallmark pathways frequently altered in cancer [61], 21 non-redundant cancer and immune related pathways obtained from the IPA Knowledgebase v6.8, and a number of additional oncogenic and tumor immune-related pathways including Hypoxia/Adenosine Immune Cell Suppression, Immunogenic Cell Death, NOS1 Signaling, PI3Kgamma signaling and the SHC1/pSTAT3 pathway as described by Lu and colleagues in 2017 [62], mechanical barrier genes as defined by Salerno and colleagues in 2016 [63], a proliferation gene signature described by Miller and colleagues in 2016 [64] and genes upregulated by MAPK mutation as described by Bedognetti and colleagues in 2017 [65].

### Enrichment of cell type abundance

To investigate the cellular composition of the TME, we considered multiple deconvolution methodologies that employ a system of linear equations to assign weighted sums to genes based on the contribution of different cell types to a given gene’s expression, including CIBERSORT [66], EPIC [67], TIMER [68], quanTIseq [69] and xCell [70]. For this study, we utilized xCell in the immunedeconv (v2.1.0) R package [71], which computes abundance ssGSEA-derived scores for 64 cell types, as it was the least restrictive method with respect to handling log transformed quantile normalized data.

### Tumor mutational burden (TMB) analysis

TMB estimates for tumors comprising the OV1 data set were originally computed from the multi-center mutation calls of the TCGA pan-cancer atlas [72] using TCGA whole-exome sequencing data. The mutation calls were obtained from the GDC knowledge base maintained at the NCI Genomic Data Commons (https://gdc.cancer.gov) by Niknafs and colleagues [73]. TMB values computed from the nonsynonymous mutations, as well as the corresponding “loss-prone” and “persistent” TMB calculations performed by Niknafs, et. al. [73], were obtained from the supplemental files of that publication.

### Assignment of HGSC molecular subtypes

Tumors were assigned to HGSC molecular subtypes: Immunoreactive (IMR), Differentiated (DIF), Proliferative (PRO) and Mesenchymal (MES) using the consensusOV 1.16.0 R package [74]. ConsensusOV is a random forest classifier trained on HGSC tumors with strong subtype agreement between different subtyping methods [74]. For each tumor, the classifier computes margin scores that reflects the degree of confidence with which the tumor belongs to each of the four subtypes (IMR, DIF, PRO and MES). In practice, the tumor is assigned to the subtype corresponding to the highest margin score. However, in instances where a tumor’s margin scores are similar across subtypes, the accuracy of subtype assignment is questionable. To address this, we ran the classifier on each data set six times, and compared each tumor’s margin scores for inconsistencies in subtype assignment. For each tumor, the margin scores for each subtype were averaged across the six runs and compared. When the difference between a tumor’s top two subtype margin scores was < 0.1 (i.e., a 10% difference in confidence) the tumor was not assigned to a subtype. Accordingly, 13.11% of tumors in the combined training and test groups were deemed ineligible for downstream subtype analyses.

### Statistical analyses

Survival analyses, including Cox proportional hazards regression and logrank tests, were performed as implemented in the R survival package (https://cran.r-project.org/web/views/Survival.html) or SigmaPlot 12.0. Overall survival (OS) was defined as the time of diagnosis to death or last clinical follow up at 8 years. This standardized threshold was employed for two reasons: 1) to more accurately compare survival dynamics across different patient populations, particularly when data sets included > 20 years survival for some patients, and 2) to minimize the effects of age-specific mortality. In Cox models, variables were treated as categorical or continuous as described in table footnotes, and Wald test p-values were reported with hazard ratios and 95% confidence intervals. Significant differences between variable distributions were assessed by t-test or the Mann–Whitney rank sum test when test for normality (Shapiro–Wilk) failed.

## Results

### CONSTRU identifies genes that alter the prognostic performance of the CYTscore signature

Gene expression profiles of HGSC were analyzed for associations between immune effector cell function and patient OS. We used the previously published gene signature, CYTscore [31], to assess the potential survival benefit of anti-tumor immunity in multiple large, independent HGSC patient populations (Table 1). First, cases were stratified into tertiles based on tumor CYTscore values, and survival differences among the tertiles were assessed by logrank test (Additional file 1: Fig. S1A–F). Cox models were then used to evaluate CYTscore prognostic effect when analyzed as a continuous variable while adjusting for patient age, International Federation of Gynecologic Oncology (FIGO) stage and tumor debulking status (Additional file 1: Fig S1G). While associations were not consistently observed, higher CYTscore values tended to correlate incrementally with improved OS, reminiscent of previous reports associating immune gene signatures with HGSC survival [49, 52, 75, 76].

**Table 1:** HGSC expression profiling data sets

	TRAINING			TEST		
	OV1	OV2	OV3	OV4	OV5	OV6
Tumor No.	431	227	221	174	260	212
Average age (range)	59.7 (30–89)	60.4 (23–80)	61.8 (38–85)^a^	63.2 (24–89)	NA	59.3 (35–80)
No. FIGO stage 1–2	35	19	4	8	0	21
No. FIGO stage 3–4	396	208	215	166	260	191
No. optimal debulking^b^	183	122	77	123	103	154
No. suboptimal debulking^c^	43	79	77	48	157	57
Gen profiling platform	Affymetrix	Affymetrix	Affymetrix	Agilent	Agilent	Illumina

^a^Only 20% of cases annotated for age

^b^Optimal (< 1 cm of residual disease)

^c^Suboptimal (> 1 cm residual disease)

Multiple studies have shown that the prognostic and predictive power of immune signatures may be restricted to distinct tumor subgroups with favorable immunogenic properties [28, 64, 77]. We therefore considered the possibility that a hidden subclass of HGSC might exist for which effector cell activity would associate robustly with survival benefit. To uncover such a subtype, we developed a statistics-guided data mining approach to screen genome-wide expression profiles for genes whose expression levels impact the prognostic performance of the CYTscore. Termed CONSTRU (Computing Prognostic Marker Dependencies by Successive Testing of Gene-Stratified Subgroups), the algorithm measures how the relative expression state for each gene (e.g., low, intermediate or high) in a typical RNAseq or microarray data matrix enhances or antagonizes the statistical association between a signature score and patient survival (Fig. 1A). In its current implementation, each gene of a HGSC data matrix was used to stratify patients into tertile groups based on the gene’s relative expression level (see Methods). Then within each tertile, a multivariable Cox model was fitted to compute the significance (p-value) and directionality (hazard ratio) of the association between the CYTscore and patient survival. This analysis was performed iteratively for each gene in the matrix. As shown in Fig. 1B, the output is an array of gene tertile-based Cox model statistics that can be ranked by a parity score to enable the sorting of genes into two desirable categories:

1. Genes whose *low* expression is uniquely conditional for a significant positive association between the CYTscore and OS (i.e., “LowerT” genes).
2. Genes whose *high* expression is uniquely conditional for a significant positive association between the CYTscore and OS (i.e., “UpperT” genes).

We applied CONSTRU to two cohorts OV1 and OV2, and the results were compared for top-performing genes. In each cohort, the parity score was used to assign percentile ranks across all genes. Using different upper and lower bound percentile-rank thresholds, we identified high-ranking UpperT and LowerT genes common to both cohorts (Fig. 1C-E). For these genes, we evaluated the relationship between their gene tertiles (T1, T2, T3) and corresponding CYTscore-survival hazard ratios (HRs). The significance of these relationships was tested using an independent HGSC cohort, OV3 (Table 1). As shown in Fig. 1C, using the top 2-percentile cutoff, 4 and 11 genes were found to overlap in the OV1 and OV2 UpperT and LowerT gene lists, respectively. As designed, the HRs of the UpperT genes (left panel) were smaller (i.e., associated positively with survival) in their upper (T3) tertiles as compared to their corresponding HRs in the lower (T1) tertiles; whereas the LowerT gene HRs (right panel) were smaller in their T1 tertiles as compared to their T3 tertiles. When assessed in the independent OV3 cohort, these relationships were reproducible. The hazard ratio distributions for these genes remained significantly different between the T3 and T1 tertiles of the selected genes (UpperT genes, *P* < 0.001; LowerT genes, *P* < 0.001). Furthermore, these relationships remained significant irrespective of increasing numbers of UpperT and LowerT genes, as selected using the 4th and 6th percentile cutoffs (Fig. 1D, E). These findings support the hypothesis that in HGSC, the relative low or high expression states of certain genes are reproducibly associated with the prognostic performance of an effector cell cytolytic activity signature.

### A patient stratification signature (STRATsig) delineates transcriptomic subtypes that differ by CYTscore prognostic performance

**Fig. 2:**
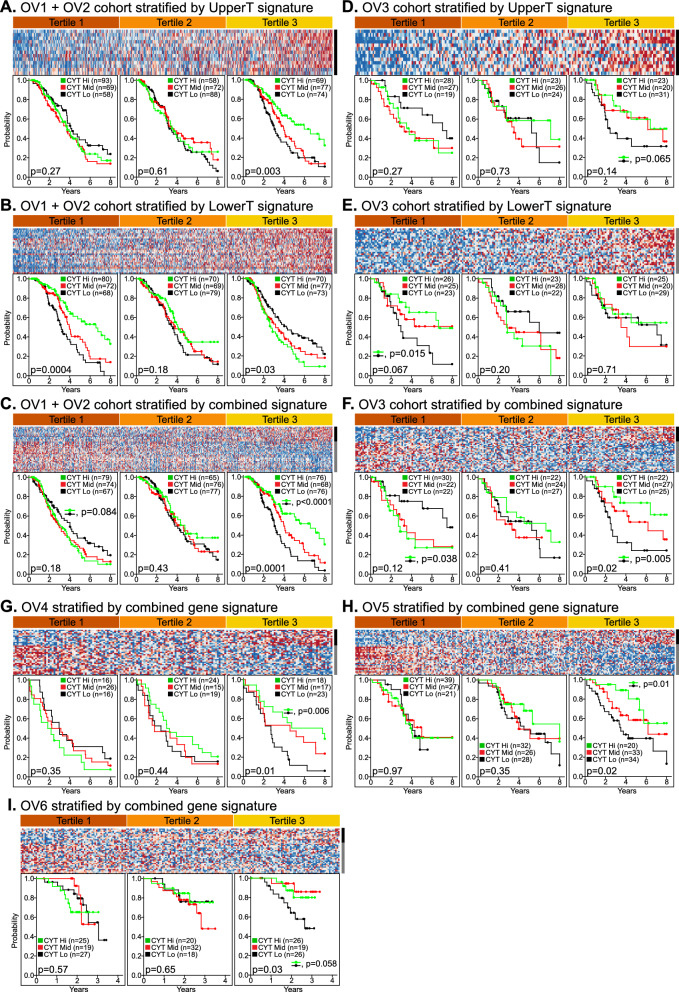
Stratification signatures and the reproducibility of tertile-specific CYTscore-survival associations. UpperT, LowerT and the combined (STRATsig) gene signatures were used to stratify cases into population tertiles for assessment of CYTscore-survival associations by Kaplan–Meier analysis. **A**–**C** Shown are results for the OV1 + OV2 combined cohort stratified by **A** the UpperT signature, **B** the LowerT signature and **C** the combined signature. **D**–**F** Results are shown for cases of the OV3 cohort stratified by **D** the UpperT signature, **E** the LowerT signature and **F** the combined signature. Similar results are shown for the validation cohorts **G** OV4, **H** OV5 and **I** OV6. CYTscore tertiles (Lo, Mid, Hi) were determined using all cases of a cohort as input. Heat maps of genes hierarchically clustered by average linkage clustering (with Pearson correlation as distance metric) are shown. Red indicates above-mean expression; blue denotes below-mean expression. Black and gray vertical bars to the right of heat maps denote UpperT and LowerT genes, respectively. Logrank p-values are shown

We next sought to integrate the unique properties of CONSTRU-selected genes for the development of a multi-gene signature for patient stratification. We first examined the internal correlation structure of UpperT and LowerT gene groups. The majority of genes showed positive correlation with one another within groups, while negative correlation predominated between groups. For each tumor, we leveraged this correlation structure by computing UpperT and LowerT gene signature scores, defined as the mean log_2_ expression of genes comprising each signature. We ultimately selected UpperT and LowerT genes defined by the 4th-percentile cutoff (Fig. 1D; n = 13 UpperT genes, n = 27 LowerT genes) on the basis of optimizing the tradeoff between selecting the highest-ranking genes and retaining a sufficient number of genes to allow robustness against error inherent in individual gene measurements. The UpperT and LowerT signatures were evaluated in the training and test cohorts for their abilities to stratify patients into tertiles that differed by CYTscore prognostic power. In the combined training data set (OV1 and OV2 combined), higher CYTscore values associated significantly with longer overall survival only in the upper tertile of patients stratified by the UpperT signature (Fig. 2A) and in the lower tertile of patients stratified by the LowerT signature (Fig. 2B). In the test cohort (OV3), these same relationships were observed (Fig. 2D, E), illustrating the reproducibility of the signatures.

The two signatures were then combined into a single stratification signature by subtracting the LowerT score from the UpperT score. As shown in Fig. 2C, F, this combined signature (termed STRATsig) stratified cases of the training and test cohorts into tertiles that differed markedly with respect to CYTscore prognostic significance. Higher STRATsig scores defined a patient upper tertile (T3) conditional for a significant positive CYTscore-survival association, while no positive association was observed in STRATsig tertiles T1 and T2, though an inverse CYTscore-survival association was observed in T1. To determine the reproducibility of these observations, we examined three additional independent HGSC cohorts for which expression profiles and clinical outcomes were available (OV4, OV5 and OV6). For each cohort, we allowed the expression data to be processed and normalized according to the authors’ published methods. We then computed their STRATsig and CYTscore tertiles and tested for CYTscore-survival associations. In all three cohorts, CYTscore achieved statistical significance only in patients comprising STRATsig (S)-T3 (Fig. 2G–I). We further confirmed this finding by using individual gene surrogates for STRATsig and CYTscore in an analysis involving > 3,700 HGSC cases from the Ovarian Tumor Tissue Analysis (OTTA) consortium study [52, 53] (Additional file 1: Fig. S2). Together, these findings confirm the broad reproducibility of the S-T3 CYTscore-survival relationship.

**Fig. 3:**
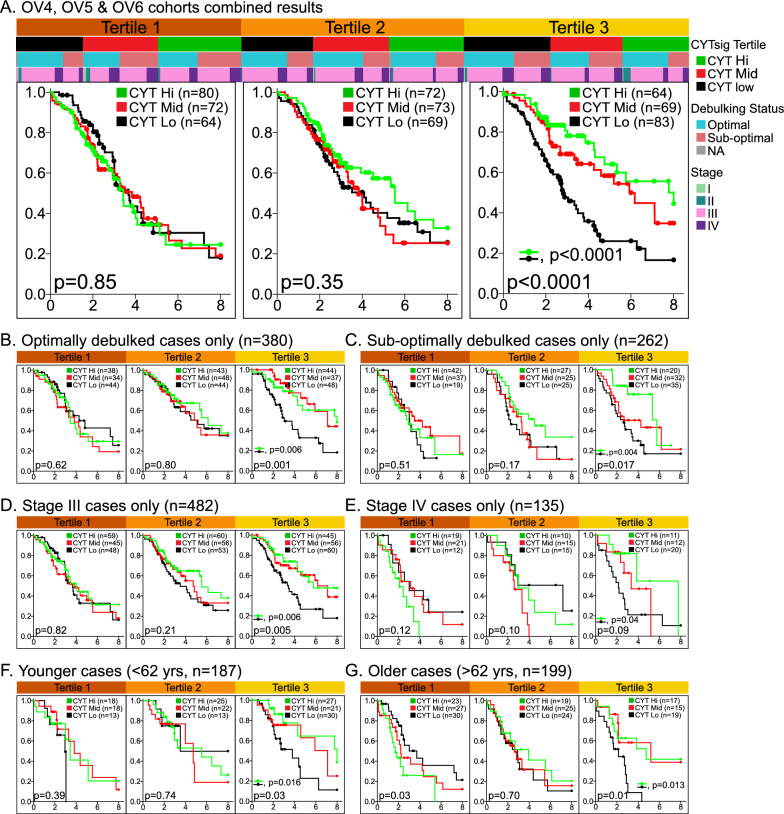
The S-T3 CYTscore-survival association is independent of debulking status, stage and patient age. **A** The integrated test group comprising cohorts OV4, OV5 and OV6 was used to assess relationships between tertile-specific CYTscore-survival associations and known prognostic factors. Cross-tertile Kaplan–Meier plots are shown for cases categorized as **B** optimally debulked, **C** sub-optimally debulked, **D** FIGO stage III, **E** FIGO stage IV, **F** younger age (< 62) and **G** older age (> 62). Logrank p-values are reported. With respect to treatments, all patients of each cohort received standard of care platinum based chemotherapy and surgery, while none received immunotherapy. Half of OV6 patients received concurrent bevacizumab (a VEGF inhibitor); however, this did not impact performance of STRATsig or CYTscore when investigated as a co-variable

Next, we tested whether the CYTscore-survival association of S-T3 was influenced by conventional prognostic factors. We merged STRATsig and CYTscore assignments and clinical annotations of OV4, OV5 and OV6 (Fig. 3A), and examined CYTscore in the STRATsig tertiles after separating cases based on FIGO stage, surgical debulking status and patient age (Fig. 3B–G). In S-T3, the CYTscore remained significantly associated with OS independent of stage, debulking status and age. In parallel, we performed multivariable Cox regression analysis to evaluate CYTscore as a continuous variable in the presence of stage, debulking status and age. For this analysis we used the ComBat empirical Bayes method [47] for batch correction to combine tumor expression profiles of OV4, OV5 and OV6 into one data set (n = 646). We used this integrated data set (referred to hereafter as the test group) to re-compute CYTscore and STRATsig values. Consistent with initial findings, the CYTscore remained significantly associated with survival in S-T3 (*P* < 0.0001) after adjusting for stage, debulking status and age, but did not achieve significance in the first or second STRATsig tertiles (*P* = 0.64 and *P* = 0.14, respectively) (Table 2). Sub-optimal surgical debulking was the only variable that remained significantly associated with poor survival in all three STRATsig tertiles.

**Table 2:** Multivariable Cox regression for associations with overall survival (test group)

	STRATsig T1 (n = 216)			STRATsig T2 (n = 214)			STRATsig T3 (n = 216)		
	No. (%)	HR (95% CI)	P-value	No. (%)	HR (95% CI)	P-value	No. (%)	HR (95% CI)	P-value
Age < 62 year	45 (21)	Ref.	–	81 (38)	Ref.	–	75 (35)	Ref.	–
Age ≥ 62 year	72 (33)	0.70 (0.42–1.15)	0.15	70 (33)	1.48 (0.92–2.36)	0.1	43 (20)	0.74 (0.42–1.29)	0.28
Low (stage I–II)	10 (5)	Ref.	–	7 (3)	Ref.	–	12 (6)	Ref.	–
High (stage III–IV)	206 (95)	0.17 (0.02–1.23)	0.08	207 (97)	0.45 (0.06–3.29)	0.43	204 (94)	0.37 (0.09–1.54)	0.17
Optimal debulking	117 (54)	Ref.	–	134 (63)	Ref.	–	129 (60)	Ref.	–
Suboptimal debulking	97 (45)	1.85 (1.23–2.80)	0.003	78 (36)	2.52 (1.66–3.84)	< 0.0001	87 (40)	2.02 (1.27–3.21)	0.003
CYTscore, continuous	Ref.	0.96 (0.82–1.13)	0.64	72 (34)	0.91 (0.79–1.03)	0.14	64 (30)	0.70 (0.60–0.81)	< 0.0001

Finally, we considered the impact of HGSC molecular subtypes – Immunoreactive (IMR), Differentiated (DIF), Proliferative (PRO) and Mesenchymal (MES). While STRATsig tertiles varied with respect to molecular subtype composition (Additional file 1: Fig. S3A–F), the CYTscore survival associations could not be explained by molecular subtype (Additional file 1: Fig. S3G, H). These findings suggest that the CYTscore-survival association observed in S-T3 occurs independent of currently understood prognostic variables in HGSC.

### STRATsig tertiles differ by pathways of immune suppression and evasion

**Fig. 4:**
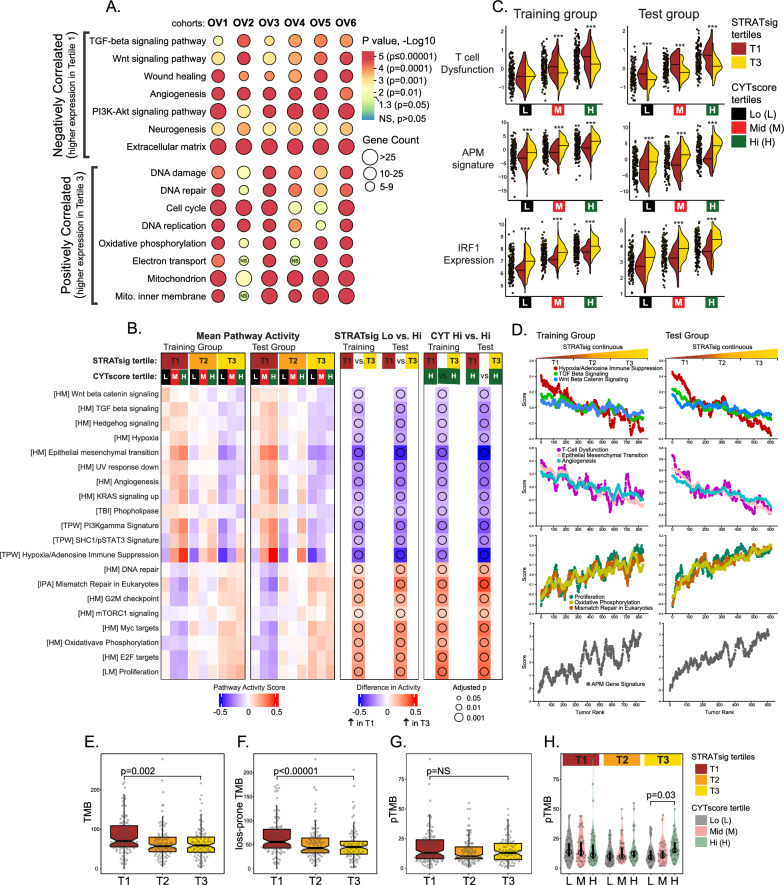
STRATsig tertiles differ by pathways of immune suppression, T cell dysfunction, antigen presentation and TMB. **A** Pathway enrichment analysis using the DAVID knowledgebase. For each cohort, the top 2% of STRATsig positively or negatively correlated genes were analyzed. **B** Pathway activation scores were computed by single-sample gene set enrichment analysis in the integrated training (OV1-OV3) and test (OV4-OV6) groups. The mean pathway activity heat map shows the average of pathway activity scores for each CYTscore group (Lo, Mid, Hi) within each STRATsig tertile. Red indicates higher pathway activity; blue denotes lower pathway activity. The 20 pathways with significant activity differences between STRATsig T1 and T3, or CYTscore Hi groups within STRATsig T1 and T3, specifically, are shown. Right 2 panels: blue reflects higher activity in T1; red indicates higher activity in T3. **C** Comparison of measures of T cell dysfunction, APM signature and IRF1 gene expression between matched CYTscore groups belonging to STRATsig tertiles T1 and T3. *P 0.01; **P 0.001; ***P < 0.001 **D** Tumors were ranked by STRATsig (left to right, ascending), and the mean pathway activation score was computed within a sliding window of n = 40 with a slide increment of + 1. The running mean score for select pathways is shown plotted across the stratified training and test populations. **E–H** In OV1, the tertile-specific distributions of tumor mutational burden (TMB) defined as **E** conventional TMB, **F** loss-prone TMB or **G** persistent TMB (pTMB) are shown. **H** pTMB distributions are shown as a function of CYTscore groups within STRATsig tertiles. Mann–Whitney U test p-values are shown

We reasoned that the CYTscore-survival association which exists in S-T3 but not in lower tertiles, may reflect tertile-dependent differences in immune regulatory potential. Therefore, we tested whether biologic pathways or processes operative in tumors might vary with respect to the tertiles. For each of the six cohorts, genes were ranked by their correlation with STRATsig values, and gene ontology enrichment analysis [59] was performed on the top 2% of the most positively or negatively correlated genes (Fig. 4A). In parallel, we used integrated training and test groups (i.e., OV1, OV2 and OV3 in training group (n = 880); OV4, OV5 and OV6 in test group (n = 647)) to quantify activation levels of 54 cancer-related pathways within both STRATsig and CYTscore tertiles (Fig. 4B). From these analyses, a number of significant pathways associated with immune suppression emerged as common themes that could reproducibly differentiate S-T1 and S-T3 populations. Enrichment for *TGF-β signaling*, *Wnt signaling*, and *angiogenesis* was associated with higher expression in S-T1 in all six cohorts (Fig. 4A). Similarly, pathway activation scores for *TGF-β, Wnt, angiogenesis,* and *hypoxia/adenosine-mediated immune suppression* were significantly higher in S-T1 relative to S-T3 (Fig. 4B, D). Importantly, these differences remained significant when comparing the CYT-Hi fractions of S-T1 and S-T3 (Fig. 4B, right panel). The CYT-Hi groups, which have uniformly high CYTscore values that do not differ across STRATsig tertiles (Additional file 1: Fig. S4A, B), display significant survival differences, particularly when comparing S-T1 CYT-Hi to S-T3 CYT-Hi, where the average median survival times are 3.2 years and 7.2 years, respectively (Additional file 1: Fig. S4C, D). We reasoned that the inferior survival of S-T1 CYT-Hi may reflect a heightened immunosuppression that potentiates a more severe immune dysfunction. To test this hypothesis, we employed a transcriptomic measure of T cell dysfunction [30] to compare the CYTscore groups of S-T1 to those of S-T3. As shown in Fig. 4C (top panels), in both training and test sets, T cell dysfunction scores were significantly higher in CYT-Mid and Hi groups of S-T1 as compared to their counterparts in S-T3. This finding associates T cell dysfunction with the reduced patient survival of the S-T1 CYT-Hi group.

Next, we assessed the enrichment of pathway activation in S-T3. Significant observations included growth and energy metabolism pathways, including *cell cycle* and *proliferation*, *DNA repair*, and *oxidative phosphorylation* (Fig. 4A, B, D). While these observations are consistent with previous reports correlating proliferation [64], genomic instability [78] and oxidative phosphorylation [79] to immune-mediated survival, how these pathways may contribute to tumor immunogenicity is not yet clear. In ovarian cancer, immunogenicity is enhanced by antigen presentation, and the transcriptional repression of antigen processing and presentation machinery (APM) genes is a common mechanism of immune evasion in HGSC [80]. As such, we compared the immunogenic potential of the CYTscore groups in S-T1 and S-T3 using an APM gene signature predictive of ICI response in melanoma [56]. The APM signature was significantly elevated in S-T3 CYTscore groups as compared to their cognate S-T1 CYTscore groups (Fig. 4C, middle panels), demonstrating an association between elevated APM expression and the immune-mediated survival benefit of S-T3 CYT-Hi. At a more granular level, IRF1, a major transcriptional activator of APM genes, was also significantly overexpressed in the CYTscore groups of S-T3 as compared to S-T1 (Fig. 4C, bottom panels), and this association extended to a number of core APM genes including TAP1, TAPBP, TAPBPL, PSMB8, PSMB9, PSMB10, PSME1 and PSME2 (Additional file 2: Table S1). Similarly, individual genes representative of immunosuppressive pathways also showed significant and reproducible differences between S-T1 and S-T3 (Additional file 1: Fig. S5), particularly with respect to overexpression in S-T1 CYT-Hi relative to S-T3 CYT-Hi. Notably, this included TGF-β (TGFB1, TGFB3), drivers of adenosine-mediated immune suppression (NT5E (CD73), ENTPD1 (CD39)), Wnt pathway ligands (WNT7A, WNT4), IL10 and drivers/markers of cancer-associated fibroblast activation (INHBA, WNT7A, TGFB1, FAP, PDGFRA, PDGFRB) [81–83] (Additional file 2: Table S1).

Together, these findings suggest that tumors comprising the lower STRATsig tertile are molecularly configured toward a hyper-immune suppressed state, marked by the parallel activation of multiple immunosuppressive pathways with concurrent T cell dysfunction and impaired antigen presentation. By contrast, tumors comprising the upper STRATsig tertile exhibit alterations consistent with a diminished immune suppression, more effective antigen presentation and reduced T cell dysfunction.

We next sought to determine if other immunogenic factors, such as tumor mutational burden (TMB) or cellular components of the tumor microenvironment (TME), vary across STRATsig tertiles. We examined the relationship between TMB and the STRATsig tertiles using the OV1 cohort, which was previously characterized by whole-exome sequencing [37]. We hypothesized that tumors which have evolved toward a hyper-immune suppressed state would be less subject to immunological constraints that would otherwise limit the accumulation of antigenic mutations. Consistent with this hypothesis, TMB levels, and the levels of *loss-prone* mutations, were significantly higher in S-T1 tumors as compared to S-T3 tumors (TMB: Mann–Whitney *P* = 0.002; Loss-prone TMB: *P* < 0.00001) (Fig. 4E, F). Niknafs and colleagues recently showed that TMB can be resolved into two immunogenic mutational classes, termed “loss-prone” and “persistent”, with differing immunological and clinical relevance [73]. Loss-prone mutations were defined as those that occur in diploid chromosomal regions and are thereby amenable to editing by cancer cells via chromosomal loss. By contrast, persistent mutations were defined as those that attract anti-tumor immune responses, but cannot be edited without lethal consequences (e.g., mutations occurring in haploid regions in linkage with essential genes) or because they exist in multiple copies that are unlikely to be simultaneously edited within a cell. Unlike loss-prone mutations, persistent mutations were shown to accumulate as tumors evolve under immune selective pressure, and the fraction of TMB consisting of persistent mutations (termed ‘pTMB’) was shown to be a more robust predictor of immunotherapy response in patients than TMB alone [73]. On this basis, we compared pTMB distributions across STRATsig tertiles and within CYTscore tertiles. While pTMB did not differ between S-T1 and S-T3 (Fig. 4G), we did observe an association with CYTscore that occurred only in S-T3 where pTMB was significantly higher in CYT-Hi tumors as compared to CYT-Lo tumors (Mann–Whitney *P* = 0.03; Fig. 4H). Thus, the survival advantage of patients in S-T3 CYT-Hi is associated not only with greater potential for cytolytic activity, but also higher pTMB.

We next investigated the cellular composition of tumors by computing abundance scores for 39 cell types (Additional file 1: Fig. S6). Notably, we found that for most of the effector and regulatory cell types examined, no significant associations with STRATsig tertiles were observed. Several cell types, however, did exhibit significant and reproducible tertile associations. Most notably, hematopoietic stem cells (*P* < *0.001*), monocytes *(P* < *0.001*), and cancer-associated fibroblasts (CAFs) (*P* < *0.001*) displayed greater abundance scores in S-T1 tumors, while Th1 CD4 + T cells (*P* < *0.001*) exhibited greater abundance scores in S-T3 tumors (Additional file 1: Fig. S6A–C). These findings suggest that certain cell populations may contribute to the STRATsig tertile phenotypes.

### STRATsig is an integrator of immunosuppressive pathways and regulates the prognostic power of multiple signatures of anti-tumor immunity

**Fig. 5:**
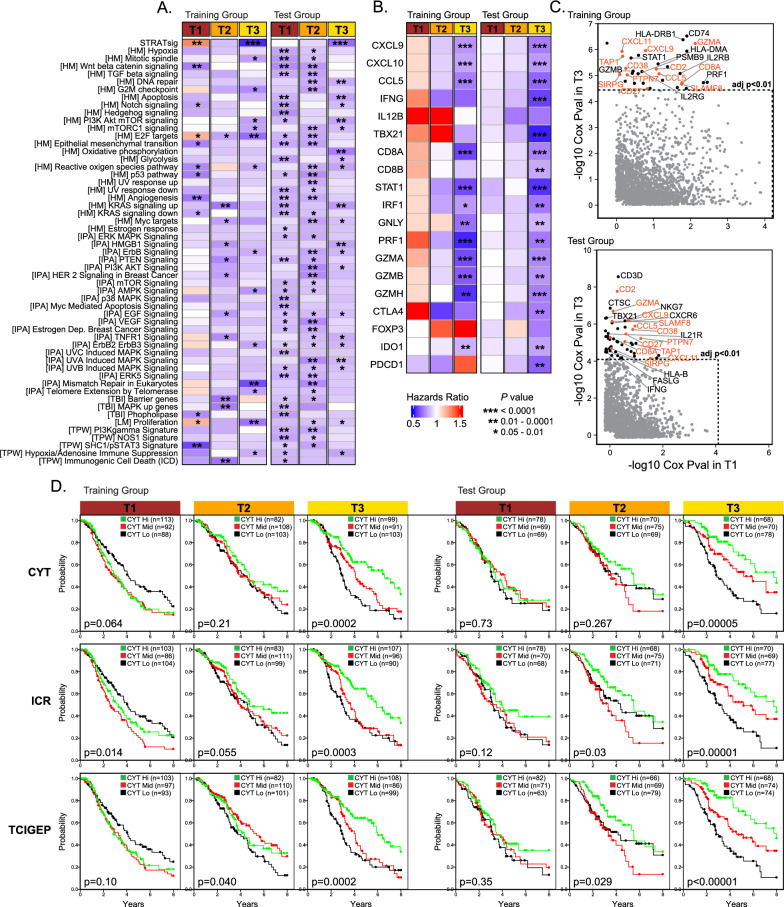
Analysis of pathway-based tertiles and survival associations using alternate immune genes and signatures. **A** Tertile reconstruction by pathways. Pathway activation scores were used to stratify cases into tertiles, and the significance of the CYTscore-survival associations within these tertiles was assessed by multivariable Cox regression. Results were compared to that of STRATsig T3 (top). **B** CYTscore deconstruction and alternative immune markers. Genes comprising the ICR gene signature were assessed for survival associations in STRATsig tertiles by Cox regression. **C** All profiled genes comprising the training and test groups were analyzed for survival associations by Cox regression in the S-T1 and S-T3 populations. Plotted are genes with hazard ratios (HR) < 0.80 in S-T1 or S-T3. Black and orange circles correspond to genes positively associated with survival with adjusted P < 0.01 (Benjamini-Hochberg). Orange denotes significant genes in common to both training and test groups. No significant genes were identified in the S-T1 population. **D** Within STRATsig tertiles, CYTscore-survival associations were compared to the survival associations observed for the ICI-predictive immune signatures, ICR and TCIGEP

Given the CYTscore-survival association specific to S-T3, we questioned which was more important: the stratification of patients by STRATsig, or the stratification of patients by the activation scores of one or more immunosuppressive pathways. We also examined the relevance of the CYTscore itself, in light of other known prognostic and predictive immune effector genes [84, 85]. In both the training and test groups, CYT-survival associations were observed for tertiles derived from pathways such as *Wnt/β-catenin signaling* (*P* < 0.01) and *Angiogenesis* (*P* < 0.01) (Fig. 5A). However, no individual pathway was able to recapitulate a tertile-specific CYT-survival association of equal or greater significance than that of S-T3 (*P* < 0.0001). With respect to the CYTscore itself, the prognostic attributes of its genes PRF1 and GZMA were not particularly unique. The expression levels of a number of genes with roles in immunological rejection [84, 85] including CD8A, STAT1, CCL5, CXCL9 and CXCL10 were also associated with survival in S-T3 with similar or greater statistical significance than that of PRF1 or GZMA (Fig. 5B). Moreover, whereas many genes involved in immune response were significantly associated with favorable survival in S-T3, no genes were identified as significantly associated with favorable survival in S-T1 after FDR adjustment (Fig. 5C). We then compared CYTscore prognostic power to that of two well-characterized immune activation signatures predictive of patient immunotherapy responses: the Immunologic Constant of Rejection (ICR) [26, 27, 86] and the T cell-inflamed Gene Expression Signature (TCIGEP) [28, 87, 88]. In the STRATsig tertiles of the training and test groups, the prognostic performances of the ICR and TCIGEP signatures were remarkably similar in effect size and significance to that of CYTscore (Fig. 5D).

Together, these observations suggest that STRATsig may function as an integrator of multiple parallel immunoregulatory pathways, rather than as a proxy of a single immunoregulatory pathway, and that alternate markers of effector cell function, in addition to PRF1 and GZMA, associate with patient survival in the S-T3 population.

## Discussion

In this work, we present a new perspective on the immunological control of high grade serous ovarian cancer, which our findings suggest depends not only on measures of infiltrating effector cells and tumor mutational burden, but also on a continuum of tumor-intrinsic immunoregulatory signaling. We demonstrate that the latter can be quantified by a patient stratification signature that serves as a determinant of the potential for immune-mediated survival benefit. Using population tertiles to dissect the meaning of this signature, we found that approximately one-third of HGSC tumors are molecularly configured toward a hyper-immune suppressed state (S-T1), characterized by heightened activation of multiple immunosuppressive pathways, with concurrent T cell dysfunction, and a reduced potential for antigen presentation. By contrast, the one-third of tumors comprising the opposite end of the spectrum (S-T3) exist in a state of diminished immunosuppressive signaling, which coincides with a more intact antigen presentation, a reduced T cell dysfunction, and an immune-mediated patient survival benefit (i.e., CYTscore) that positively correlates with persistent TMB.

In comparisons of S-T1 vs. S-T3, as well as S-T1 CYT-Hi vs. S-T3 CYT-Hi, we observed significantly greater activation scores for TGF-β, Wnt/β-catenin and CD73/CD39 adenosine-mediated immunosuppression pathways in S-T1 tumors. This association was further accompanied by significantly higher T cell dysfunction scores and significantly lower expression of antigen presentation and processing genes in S-T1. In HGSC, activation of the TGF-β and Wnt/β-catenin signaling pathways are known to produce pleiotropic effects. Not only do they promote tumor progression by inducing epithelial-mesenchymal transition (EMT) [89, 90], stem cell self-renewal [91] and chemoresistance [92], but they also drive T cell dysfunction and exclusion by (i) regulating lymphocyte differentiation, expansion and survival [93, 94], and (ii) by promoting Treg accumulation, dendritic cell tolerance, and myeloid immune-suppressive functions [95, 96]. TGF-β has also been shown to directly down-regulate GZMA and PRF1 (i.e., the components of the CYTscore) in cytotoxic T cells [97]. CD73 and CD39 are ectonucleotidases that convert ATP to free adenosine. The accumulation of extracellular adenosine in the TME impairs the recruitment and activation of CD8 + T cells and NK cells and promotes the immunosuppressive functions of tumor associated macrophages [98]. In HGSC, this pathway has been reported to promote tumor immune escape and is associated with poor prognosis [99, 100].

When we analyzed the relative abundance estimates of different cell types in STRATsig tertiles, we found that the fraction of cancer associated fibroblasts (CAFs) was significantly higher in S-T1 as compared to S-T3. CAFs are known to have an immunomodulatory secretome that can suppress effector cell function by inhibiting the activation and survival of cytotoxic T cells and by promoting the recruitment and activation of immunosuppressive myeloid cells and Tregs [101]. Interestingly, CAFs, which are intimately linked to ovarian cancer progression [102], are frequently activated by TGF-β [103–105] or Wnt [106] signaling pathways in HGSC and other tumor types, and this process can be driven by WNT7A [83], a LowerT gene belonging to our STRATsig signature.

In S-T3, where we observed a significant reduction in the immunoregulatory pathways described above, we also observed a significant increase in the Th1 CD4 + T cell fraction as compared to S-T1. This increase was similarly observed in S-T3 CYT-Hi relative to S-T1 CYT-Hi and links a Th1-polarized CD4 + T cell population with patient survival benefit that is associated with heightened effector cell activity. This finding could reflect the importance of a helper T cell population in the recruitment and activation of effector cells capable of restraining HGSC clinical progression. Recently, it was reported that a CD4^+^/CD25^+^/FOXP3^−^ T cell population with an exhausted Th1-like polarization comprising up to 13% of CD4 + TIL, was highly correlated with HGSC progression-free survival [107]. Whether our finding reflects the activity of this cell subtype warrants further investigation. In another recent report, a tumor-reactive progenitor CD8^+^/TCF1^Lo^ tissue-resident memory T cell population (TRM_stem_ cells) was shown to be the predominant CD8 + T cell subtype associated with HGSC survival and sustained antigen recognition [19]. An understanding of how these cells function in the context of the STRATsig tumor backgrounds differentiated in our study could yield important clinical insights.

In S-T3, but not other tertiles, we observed that pTMB was significantly higher in CYT-Hi as compared to CYT-Lo, thus linking an immune-mediated survival advantage in HGSC to higher neo-antigen load, consistent with previous observations [108]. By contrast, the reduced survival experienced by S-T3 CYT-Lo patients may result from an ineffectual anti-tumor immunity owed, in part, to suboptimal neo-antigen load.

These findings, together with the immunoregulatory differences described above, corroborate the view that tumors of the S-T3 class share a *hypo-*immunosuppressed and antigenic molecular composition permissive of immunologic tumor control. By extension, it is plausible that this subpopulation of patients may share a propensity for response to immune checkpoint blockade. While immune checkpoint inhibitors (ICI) have proven efficacious for a number of solid tumor types [5–9], clinical trials in HGSC have been widely disappointing [11]. However, it has been reported that patients who do respond may experience durable responses of 6 months or longer [13–15]. A biomarker that can distinguish these patients at diagnosis could more accurately guide treatment strategies. Several lines of evidence suggest that patients of the S-T3 class may have a heightened potential for ICI response.

First, the Immunologic Constant of Rejection (ICR) [26, 27, 86] and the T cell-inflamed Gene Expression Signature (TCIGEP) [28, 87, 88] are well-characterized immune activation signatures that are both prognostic of cancer survival outcomes and predictive of ICI clinical efficacy in multiple tumor types. In our study, we found that both signatures were highly significantly associated with survival in S-T3 patients, but not those of S-T2 or S-T1. Second, we found that an APM signature [56] predictive of ICI response in melanoma, was significantly elevated in tumors of S-T3 as compared to S-T1 across all CYTscore groups. Third, NikNafs et. al. recently demonstrated that a new measure of TMB based on ‘persistent’ mutations (i.e., pTMB), is a more significant predictor of immunotherapy response than TMB alone [73]. In our study, pTMB was associated with CYTscore only in S-T3, and was significantly higher in the survival-advantaged CYT-Hi population.

The discovery of STRATsig was enabled by a new algorithm, CONSTRU, designed to facilitate the discovery of previously unrecognized patient subpopulations that differ with respect to the performance of a classifier. While our application was specific to a prognostic immune signature, the algorithm is amenable to any type of continuous or catagorical variable, including individual genes inherent to the data set that may or may not be associated with the outcome/attribute in question when assessed at the whole-population level. In this sense, any gene or gene signature (eg., expression, methylation, copy number, proteomic) believed to reflect a pathway related to cancer progression could be used by CONSTRU to define a population of patients for which it may be most applicable as a prognostic biomarker. Furthermore, important biological insights into the nature of the tumors comprising that population may be revealed by the analysis of the genes most correlated with the stratification signature.

A central function of CONSTRU is to measure how gene expression levels influence the effect of a gene or gene signature on survival. Conceptually, this is akin to measuring variable interactions in Cox proportional hazards models, which was the approach used in a recent report to uncover genes that contribute to T cell dysfunction [30]. In CONSTRU, gene expression is binned into quantiles to enable control of the size of patient subpopulations where interactions may be discovered. In our study, the use of gene tertiles allowed the standardization of relative expression increments (i.e., low, intermediate and high) across all genes, resulting in the delineation of patient subpopulations of equal and adequate size and power for downstream statistical comparisons. Future enhancements to CONSTRU will include refinements to the approach, such as incorporating Cox modeling for interactions in the gene ranking process and the use of methods to define optimal cutpoints for each gene and to select stratifcation signature quantiles optimized simultaneously for the interaction in question and the population under study.

There are several limitations to this work. In HGSC, the tumor compartment in which TILs reside (i.e., intraepithelial versus stromal) is a strong determinant of TIL prognostic power, with intraepithelial TIL being most associated with survival [109–111]. In the tumor expression profiles analyzed in our study this information is lost. Thus, the CYTscore likely reflects the admixture of intraepithelial and stromal TIL which could obscure its prognostic power. Whether or not TIL compartment bias is related to the different CYTscore-survival associations observed between S-T1 and S-T3 remains to be determined. Intrinsic TIL heterogeneity is another limitation. T-cell hot and cold micro-compartments are known to co-exist within HGSC tumors [112], and this heterogeneity impacts the TIL survival association. In a multi-center survival study involving more than 3,000 HGSC cases [113], substantial heterogeneity in CD8 + TIL was observed across multiple core samples from the same patient. The significant effect of CD8 + TIL on survival observed in the study was determined by a scoring system that, for each patient, identified TIL hotspots in the available cores and assigned a score equivalent to the maximum TIL score (i.e., number of TIL per high-power field counted within a hotspot) observed among all the cores for a patient. In our study, multiple cores per patient could not be assessed, as only a single tumor specimen per patient was profiled for gene expression. Therefore, it is likely that some cases in our study are innately T-cell hot, but misclassified as CYT-Lo due to the chance selection of a T-cell excluded tumor specimen. Finally, tertiles as a means to study patient populations, though advantageous for research purposes, may lack precision for clinical applicability. Future work to address these limitations are prerequisite for clinical translation.

Finally, additional questions linger. If patients classified as S-T3 were to prove more responsive to immune checkpoint inhibitors, what then could be offered to patients of S-T1 and S-T2, where immunosuppressive pathways dominate? New research efforts aimed at targeting alternative immunosuppressive pathways (other than the PD-1/PD-L1 axis) may be key to reaching these patients. Indeed, work is already underway to therapeutically inhibit the WNT/β-catenin [114] and TGF-β [115] pathways in HGSC.

## Conclusion

In summary, we show that variations within the molecular background of HGSC are significant determinants of immune-mediated patient survival. The potential for this immune control can be measured, in part, by a patient stratification signature that reflects the functional output of parallel regulatory pathways in immune suppression, evasion and dysfunction. At one end of this continuum, lies a hyper-immunosuppressed tumor phenotype where effective anti-tumor immunity is not clinically apparent; at the other, exists a phenotype of eased immunoregulation which confers a TIL/APM/TMB-linked immune-mediated survival advantage for patients. Studies aimed at elucidating the etiology of these phenotypes, and characterizing their immunogenic properties using experimental models, will be necessary first steps toward understanding their therapeutic implications.

## Supplementary Information

**Additional file 1:** Supplementary figures referenced in the main article. Figure S1. CYTscore prognostic performance in HGSC data sets. Figure S2. Surrogate gene analysis of STRATsig and CYTscore in the OTTA consortium cohort reproduces the STRATsig T3 CYTscore-survival association. Figure S3. HGSC molecular subtype composition in STRATsig and CYTscore tertiles. Figure S4. Comparison of CYTscore distributions within CYTscore groups and survival characteristics. Figure S5. Representative genes recapitulate pathway-tertile associations. Figure S6. Analysis of cell type proportions in STRATsig tertiles.

**Additional file 2:** Table S1. Differential expression of select genes between STRATsig T1 and T3 CYTscore groups.

## Data Availability

All data used in this study are publicly available as described in the Methods section. R code for the CONSTRU algorithm is accessible at GitHub (https://github.com/chifman/Constru).

## Abbreviations

CAF: Cancer associated fibroblast
CONSTRU: Computing Prognostic Marker Dependencies by Successive Testing of Gene-Stratified Subgroups
CYT: Cytolytic activity
DAVID: Database for annotation, visualization and integrated discovery
DIF: Differentiated
dMMR: Deficiency in mismatch repair
EMT: Epithelial-mesenchymal transition
EOC: Epithelial ovarian cancer
EPIC: Estimating the Proportion of Immune and Cancer cells
FIGO: International Federation of Gynecologic Oncology
HGSC: High-grade serous ovarian cancer
HR: Hazard ratio
ICI: Immune checkpoint inhibitors
IMR: Immunoreactive
MES: Mesenchymal
MSI-H: Microsatellite instability high
OS: Overall survival
PRO: Proliferative
ssGSEA: Single sample gene set enrichment analysis
STRAT: Stratification
TIL: Tumor infiltrating lymphocytes
TIMER: Tumor Immune Estimation Resource
TMB: Tumor mutational burden
Treg: Regulatory T cell
TRM: Tissue resident memory
