# Supplementary material for: A patient stratification signature mirrors the immunogenic potential of high grade serous ovarian cancers
Source: J Transl Med. 2024 Nov 20;22:1048. doi: 10.1186/s12967-024-05846-9 (PMC11577735; doi:10.1186/s12967-024-05846-9)
Supplement: Supplementary file 1 — Additional file 1: Supplementary figures referenced in the main article. Figure S1. CYTscore prognostic performance in HGSC data sets. Figure S2. Surrogate gene analysis of STRATsig and CYTscore in the OTTA consortium cohort reproduces the STRATsig T3 CYTscore-survival association. Figure S3. HGSC molecular subtype composition in STRATsig and CYTscore tertiles. Figure S4. Comparison of CYTscore distributions within CYTscore groups and survival characteristics. Figure S5. Representative genes recapitulate pathway-tertile associations. Figure S6. Analysis of cell type proportions in STRATsig tertiles. [file 12967_2024_5846_MOESM1_ESM.pdf]

A patient stratification signature mirrors the immunogenic potential of high grade serous ovarian cancers

Laurel K. Berry, Ashok K. Pullikuth, Kristen L. Stearns, Yuezhu Wang, Calvin J. Wagner, Jeff W. Chou, Janelle P. Darby, Michael G. Kelly, Raghvendra Mall, Ming Leung, Julia Chifman and Lance D. Miller

Additional File 1: Supplementary figures (Fig. S1 – S6)

Figure S1. *CYTscore prognostic performance in HGSC datasets.*

Figure S2. *Surrogate gene analysis of STRATsig and CYTscore in the OTTA consortium cohort reproduces the STRATsig T3 CYTscore-survival association.*

Figure S3. *HGSC molecular subtype composition in STRATsig and CYTscore tertiles.*

Figure S4. *Comparison of CYTscore distributions within CYTscore groups and survival characteristics.*

Figure S5. *Representative genes recapitulate pathway-tertile associations.*

Figure S6. *Analysis of cell type proportions in STRATsig tertiles.*

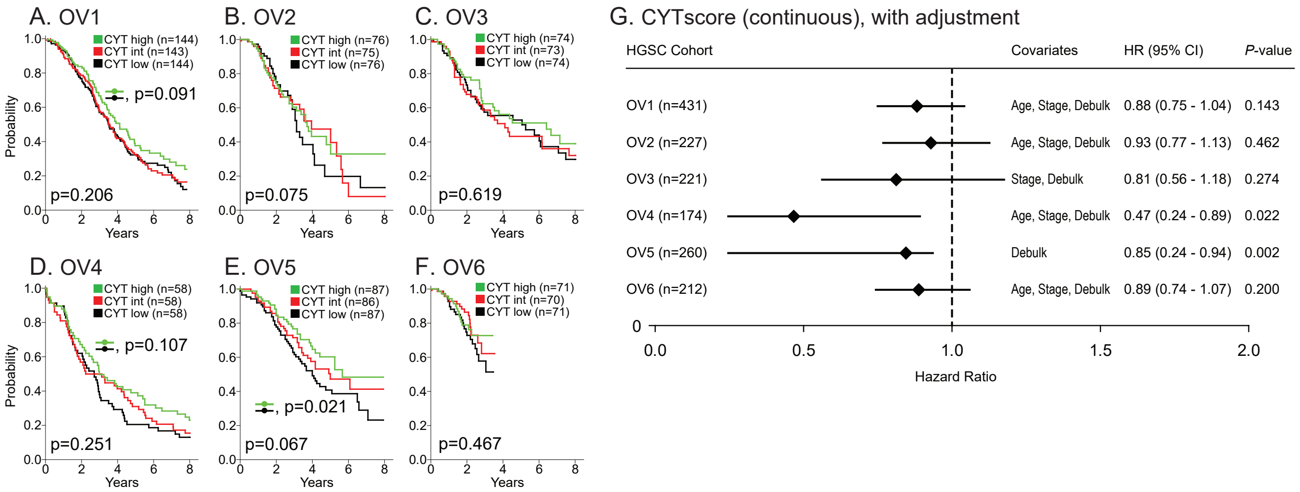

Figure S1. *CYTscore prognostic performance in HGSC datasets.* **(A-F)** In datasets OV1-OV6, tumors were stratified into tertiles based on tumor CYTscore values, and survival differences among the tertile groups were assessed by logrank test. **(G)** The CYTscore was analyzed as a continuous variable in Cox models while adjusting for patient age (continuous), International Federation of Gynecologic Oncology (FIGO) stage (I, II, III, IV) and debulking status (0, 1), when available.

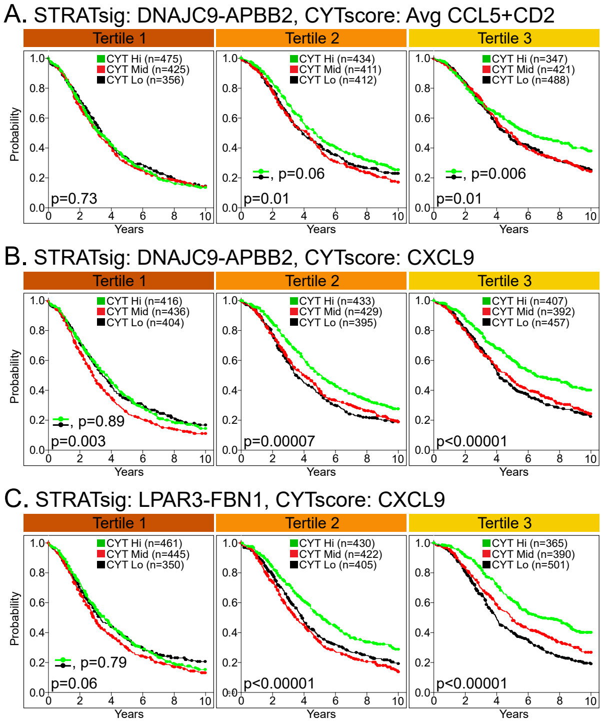

Figure S2. *Surrogate gene analysis of STRATsig and CYTscore in the OTTA consortium cohort reproduces the STRATsig T3 CYTscore-survival association.* In recent reports by the Ovarian Tumor Tissue Analysis (OTTA) consortium, FFPE tumor specimens and corresponding clinical data from 3,769 HGSC patients were collected at 20 sites across multiple countries, processed using standardized protocols, and analyzed for the expression of 513 informative genes using the NanoString n-Counter mRNA quantitation platform (Millstein, et. al., PMCID: PMC7484370; Talhouk, et. al., PMCID: PMC7572656). In this data set (GEO accession GSE132342), the reproducibility of the observed STRATsig and CYTscore associations was evaluated by proxy using individual genes comprising the signatures, as well as genes with greatest correlation to the signatures. Included among the 513 genes profiled were the STRATsig UpperT gene, DNAJC9, and the LowerT gene, APBB2. In our training and test group data sets, the average correlation between these genes and STRATsig were 0.26 and -0.45 for DNAJC9 and APBB2, respectively. While the genes comprising the CYTscore (GZMA and PRF1) were not represented in the study, the genes CCL5 and CD2 were included. In both our training and test groups, CCL5 and CD2 ranked within the top five genes most positively correlated with CYTscore, with average correlations to CYTscore of 0.91 and 0.90, respectively. **(A)** Using the ratio of DNAJC9 to APBB2 as a proxy to approximate the STRATsig vector, and the geometric mean of CCL5 and CD2 to approximate the CYTscore vector, we analyzed the OTTA data set for CYT-survival associations within the approximated STRATsig tertiles. CYTscore significance in STRATsig T1, T2 and T3 was P = 0.73 (CYT Lo vs. Hi: P = 0.32), P = 0.01 (CYT Lo vs. Hi: P = 0.06), and P = 0.01 (CYT Lo vs. Hi: P = 0.006), respectively. **(B)** As reported by the OTTA consortium (Millstein, et. al., PMCID: PMC7484370), CXCL9, a chemokine that recruits effector T cells, was identified as among the top five genes most significantly associated with overall survival in the OTTA data set. In our training and test groups, CXCL9 showed an average correlation to CYTscore of r = 0.80. Using CXCL9 as a proxy for CYTscore, CYTscore significance in T1, T2 and T3 was P = 0.003 (CYT Lo vs. Hi: P = 0.89), P = 0.00007 (CYT Lo vs. Hi: P = 0.00002), and P < 0.00001 (CYT Lo vs. Hi: P < 0.00001), respectively. **(C)** Next we reconstructed the vector for STRATsig using the two genes represented in the OTTA data set that were most positively and negatively correlated with the original STRATsig in our training and test groups. These genes, LPAR3 and FBN1, were not part of the original STRATsig gene list, but within the training and test groups, they showed average correlations to STRATsig of 0.37 and -0.54, respectively (i.e., more positively and negatively correlated to STRATsig than DNAJC9 and APBB2). Using the ratio of LPAR3 to FBN1 as a proxy for STRATsig, and CXCL9 as a proxy for CYTscore, CYTscore significance in T1, T2 and T3 was P = 0.06 (CYT Lo vs. Hi: P = 0.79), P < 0.00001 (CYT Lo vs. Hi: P < 0.00001), and P < 0.00001 (CYT Lo vs. Hi: P < 0.00001), respectively.

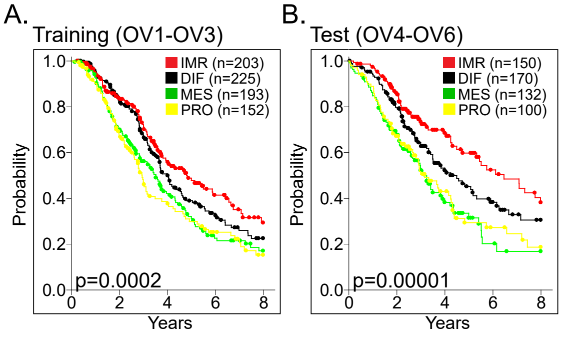

C. % subtypes within tertile groups

Training (OV1-OV3)

	DIF	IMR	MES	PRO	
STRATsig T1, CYT L	0.17	0.01	0.19	0.63	=100%
STRATsig T1, CYT M	0.24	0.11	0.54	0.11	=100%
STRATsig T1, CYT H	0.06	0.38	0.54	0.02	=100%
STRATsig T2, CYT L	0.34	0.02	0.19	0.45	=100%
STRATsig T2, CYT M	0.43	0.11	0.34	0.11	=100%
STRATsig T2, CYT H	0.11	0.66	0.20	0.03	=100%
STRATsig T3, CYT L	0.59	0.09	0.04	0.28	=100%
STRATsig T3, CYT M	0.59	0.19	0.07	0.16	=100%
STRATsig T3, CYT H	0.14	0.80	0.07	0.00	=100%

Test (OV4-OV6)

	DIF	IMR	MES	PRO	
STRATsig T1, CYT L	0.19	0.06	0.15	0.60	=100%
STRATsig T1, CYT M	0.11	0.13	0.68	0.08	=100%
STRATsig T1, CYT H	0.06	0.27	0.66	0.01	=100%
STRATsig T2, CYT L	0.39	0.00	0.08	0.53	=100%
STRATsig T2, CYT M	0.48	0.15	0.25	0.13	=100%
STRATsig T2, CYT H	0.02	0.78	0.18	0.02	=100%
STRATsig T3, CYT L	0.62	0.09	0.04	0.24	=100%
STRATsig T3, CYT M	0.58	0.30	0.05	0.07	=100%
STRATsig T3, CYT H	0.23	0.77	0.00	0.00	=100%

D. % subtypes across tertile groups

Training (OV1-OV3)

	DIF	IMR	MES	PRO
STRATsig T1, CYT L	0.06	0.00	0.08	0.34
STRATsig T1, CYT M	0.08	0.04	0.22	0.06
STRATsig T1, CYT H	0.03	0.20	0.29	0.01
STRATsig T2, CYT L	0.13	0.01	0.09	0.26
STRATsig T2, CYT M	0.17	0.05	0.16	0.07
STRATsig T2, CYT H	0.04	0.24	0.08	0.01
STRATsig T3, CYT L	0.24	0.04	0.02	0.16
STRATsig T3, CYT M	0.20	0.07	0.03	0.08
STRATsig T3, CYT H	0.05	0.34	0.03	0.00
	=100%	=100%	=100%	=100%

Test (OV4-OV6)

	DIF	IMR	MES	PRO
STRATsig T1, CYT L	0.05	0.02	0.05	0.29
STRATsig T1, CYT M	0.04	0.05	0.33	0.05
STRATsig T1, CYT H	0.02	0.13	0.35	0.01
STRATsig T2, CYT L	0.15	0.00	0.04	0.35
STRATsig T2, CYT M	0.17	0.06	0.11	0.08
STRATsig T2, CYT H	0.01	0.29	0.08	0.01
STRATsig T3, CYT L	0.27	0.05	0.02	0.18
STRATsig T3, CYT M	0.21	0.12	0.02	0.04
STRATsig T3, CYT H	0.08	0.29	0.00	0.00
	=100%	=100%	=100%	=100%

E. Training (OV1-OV3)

	DIF	IMR	MES	PRO
STRATsig T1	0.15	0.19	0.43	0.24
STRATsig T2	0.30	0.24	0.25	0.21
STRATsig T3	0.43	0.36	0.06	0.15

F. Test (OV4-OV6)

	DIF	IMR	MES	PRO
STRATsig T1	0.11	0.17	0.53	0.19
STRATsig T2	0.31	0.29	0.17	0.24
STRATsig T3	0.49	0.36	0.03	0.12

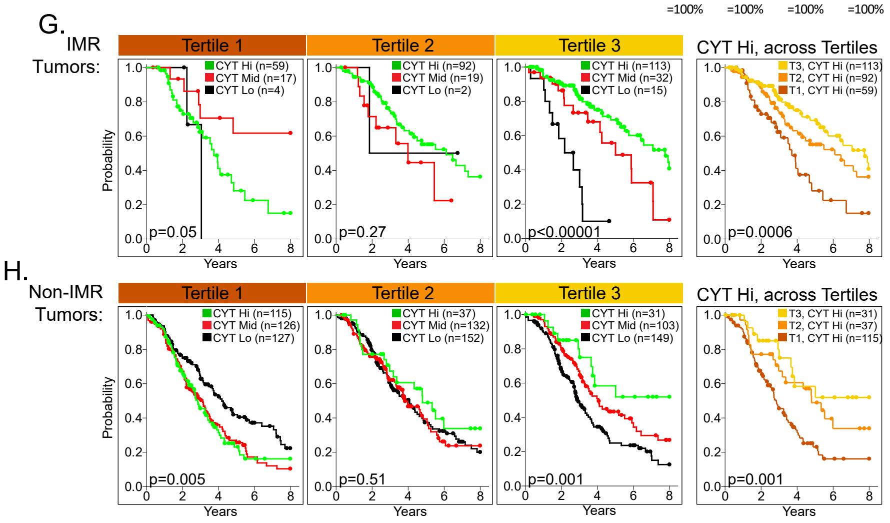

Figure S3. *HGSC molecular subtype composition in STRATsig and CYTscore tertiles.* Training and test group tumors were assigned to their respective subtypes using the consensusOV algorithm of Chen and colleagues (PMCID: PMC6207081). Similar subtype survival rates were observed when comparing **(A)** training and **(B)** test populations, with IMR and DIFF tumors having better survival than MES and PRO tumors, consistent with published observations (Konecny, et. al., PMCID: PMC4271115; Chen, et. al., PMCID: PMC6207081). **(C)** The percentage of subtypes within tertile groups, and **(D)** across tertile groups, was examined. Analysis of STRATsig tertiles subdivided by CYTscore groups showed a strong positive relationship between the IMR subtype and CYTscore, a strong negative association between PRO and CYTscore, as well as a moderately negative association between DIF and CYTscore. Analysis of subtype composition in STRATsig tertiles of the **(E)** training group and **(F)** test group showed that STRATsig T1 comprised largely of MES (43-53%) and PRO (19-24%) subtypes, while STRATsig T3 consisted mostly of DIF (43-49%) and IMR (36%) tumors; T2 tumors were more admixed, with all subtype fractions ranging from 17-31%. **(C)** In STRATsig T3, CYT Lo tumors were predominantly DIF (59-62%) and PRO (24-28%), while the CYT Hi tumors comprised mostly of IMR (77-80%) and DIF (14-23%). The relationship between IMR and the STRATsig T3 CYT-survival association was investigated. **(G)** In IMR tumors, CYTscore was associated with survival in STRATsig T3 (log-rank test P < 0.00001), but not in T2 or T1, indicating that IMR status does not fully explain the STRATsig T3 CYT-survival association. **(H)** The STRATsig T3 CYT-survival association was also observable among the non-IMR subtypes (log-rank test P = 0.001).

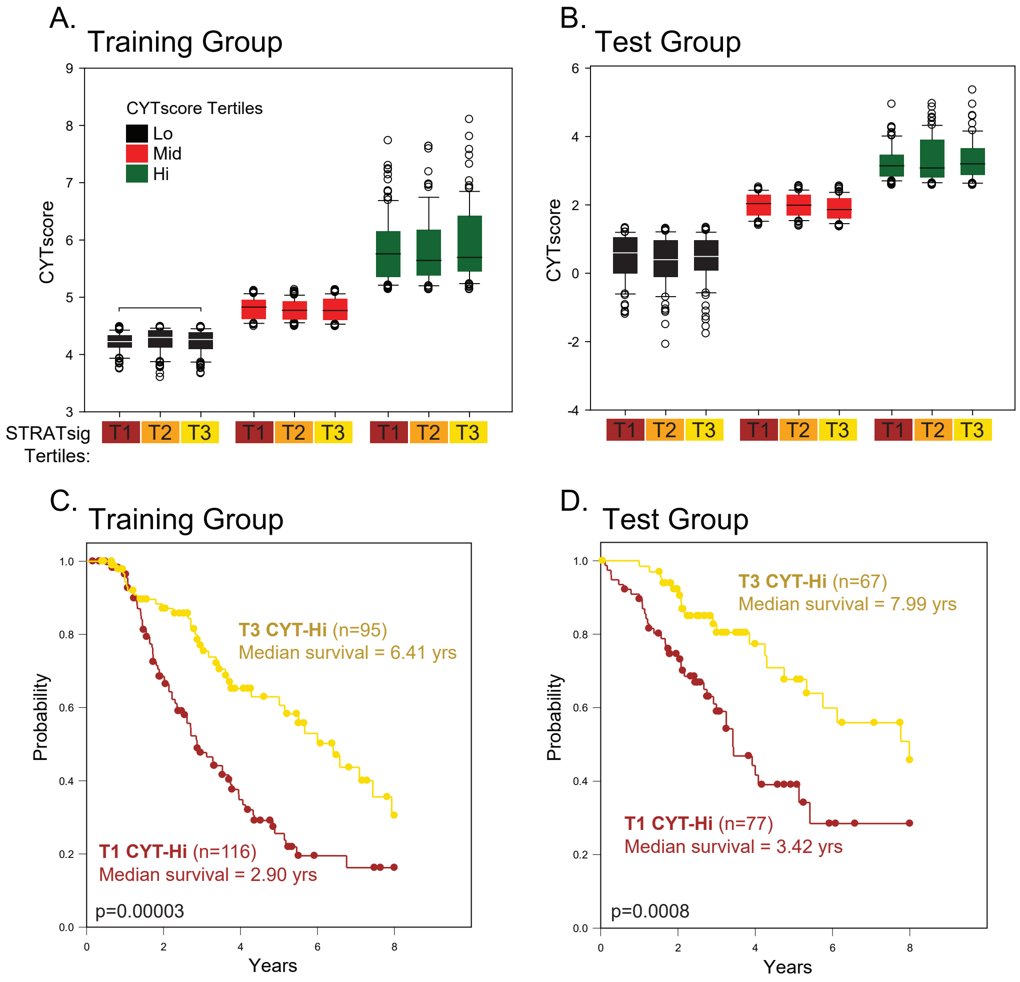

Figure S4. *Comparison of CYTscore distributions within CYTscore groups and survival characteristics.* CYTscore distributions within matched CYTscore groups are shown compared across STRATsig tertiles for **(A)** the training group and **(B)** the test group. No significant differences between (cross-tertile) matched CYTscore groups were observed. Kaplan-Meier survival curves comparing STRATsig T3 CYT Hi cases to STRATsig T1 CYT Hi cases are shown for **(C)** the training group and **(D)** the test group. Logrank p-values are reported.

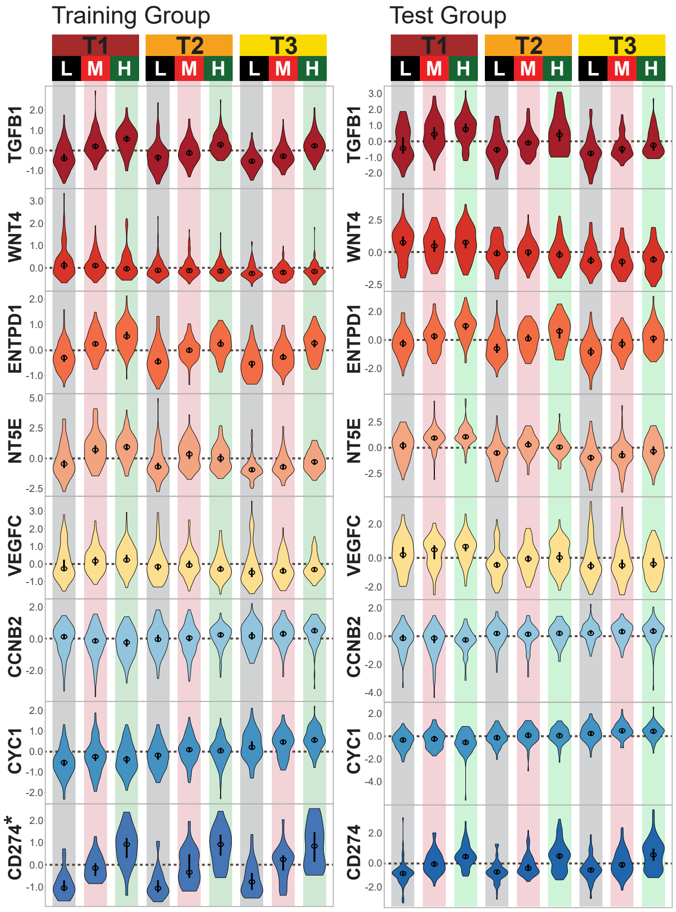

Figure S5. *Representative genes recapitulate pathway-tertile associations.* Expression distributions of representative genes associated with various pathways are shown as a function of tertile and CYTscore group. *, In the training group, expression profiles for CD274 (PD-L1) were only available in OV2.

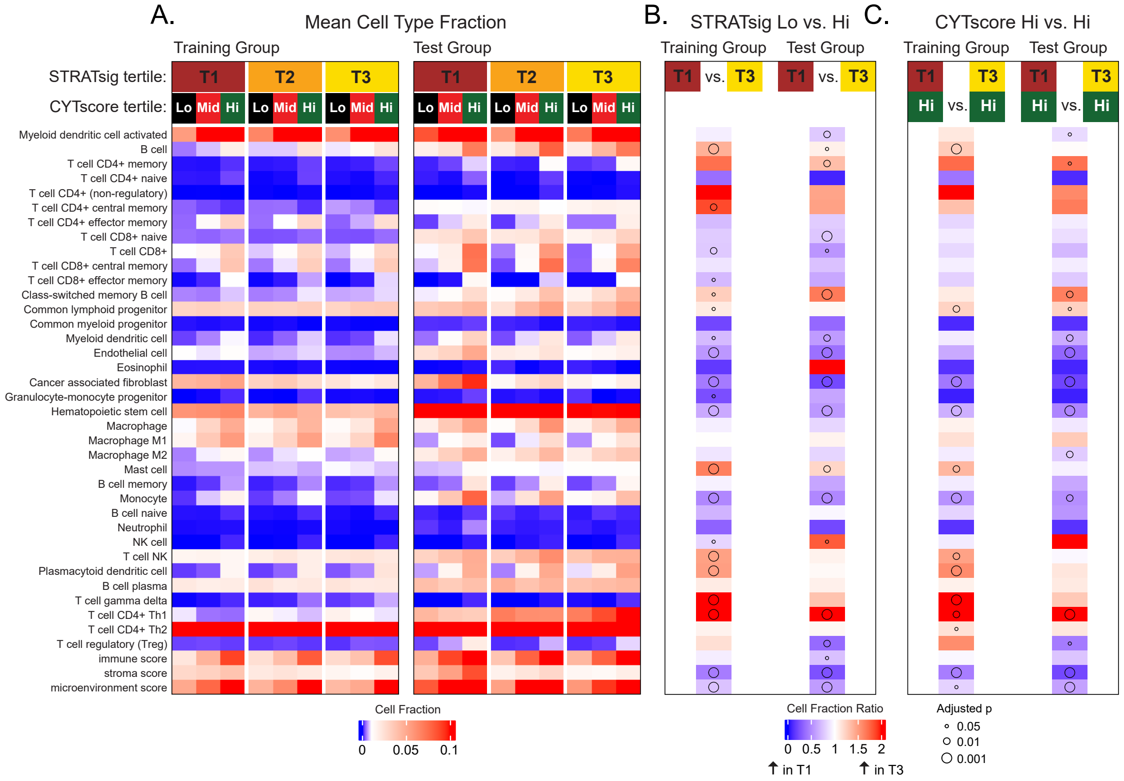

Figure S6. *Analysis of cell type proportions in STRATsig tertiles.* Cell type proportions were computed by xCell single-sample gene set enrichment analysis in the integrated training (OV1-OV3) and test (OV4-OV6) groups. **(A)** The mean cell type fraction heat map shows the average cell type fractions for each CYTscore group (Lo, Mid, Hi) within each STRATsig tertile. Red indicates higher cell fraction; blue denotes lower cell fraction. **(B)** Significant cell fraction differences between STRATsig T1 and T3; **(C)** CYTscore Hi groups within STRATsig T1 and T3, specifically. Blue reflects higher cell fractions in T1; red indicates higher cell fractions in T3. Size of the circle denotes adjusted p value with the smallest circle representing a p value of 0.05 and the largest representing a p value of 0.001.
