# Supplementary material for: A patient stratification signature mirrors the immunogenic potential of high grade serous ovarian cancers
Source: J Transl Med. 2024 Nov 20;22:1048. doi: 10.1186/s12967-024-05846-9 (PMC11577735; doi:10.1186/s12967-024-05846-9)
Supplement: Supplementary file 2 — Additional file 2: Table S1. Differential expression of select genes between STRATsig T1 and T3 CYTscore groups. [file 12967_2024_5846_MOESM2_ESM.pdf]

A patient stratification signature mirrors the immunogenic potential of high grade serous ovarian cancers

Laurel K. Berry, Ashok K. Pullikuth, Kristen L. Stearns, Yuezhu Wang, Calvin J. Wagner, Jeff W. Chou, Janelle P. Darby, Michael G. Kelly, Raghvendra Mall, Ming Leung, Julia Chifman and Lance D. Miller

Additional File 2: Supplementary table S1

Table S1. *Differential expression of select genes between STRATsig T1 and T3 CYTscore groups.*

Supplementary Table 1. Differential expression of select genes between STRATsig T1 and T3 CYTscore groups

	Test Group						Training Group						
Gene:	T1 CYT-Hi vs T3 CYT-Hi		T1 CYT-Mid vs T3 CYT-Mid		T1 CYT-Lo vs T3 CYT-Lo		T1 CYT-Hi vs T3 CYT-Hi		T1 CYT-Mid vs T3 CYT-Mid		T1 CYT-Lo vs T3 CYT-Lo		Biological Function:
	LFC	Adj. Pval	LFC	Adj. Pval	LFC	Adj. Pval	LFC	Adj. Pval	LFC	Adj. Pval	LFC	Adj. Pval	
IRF1	0.66	3.30E-07	0.60	9.34E-06	0.47	2.85E-03	0.58	9.04E-09	0.53	3.17E-07	0.73	2.89E-07	MHC class I antigen processing & presentation
TAP1	0.46	1.29E-04	0.46	1.23E-03	0.50	7.62E-03	0.58	4.98E-06	0.37	6.76E-03	0.55	3.47E-03	MHC class I antigen processing & presentation
TAPBP	0.27	8.89E-02	0.64	2.35E-04	0.66	2.44E-03	0.33	8.46E-04	0.35	1.02E-03	0.47	1.12E-03	MHC class I antigen processing & presentation
TAPBPL	0.49	2.05E-04	0.46	1.46E-04	0.27	1.09E-01	0.27	5.75E-02	0.32	4.50E-02	0.40	1.33E-02	MHC class I antigen processing & presentation
B2M	0.05	7.04E-01	0.14	2.50E-01	0.08	7.28E-01	0.06	3.48E-01	0.01	9.14E-01	0.16	1.23E-01	MHC class I antigen processing & presentation
PDIA3	0.18	9.14E-02	0.14	1.34E-01	0.18	1.66E-01	0.40	3.48E-06	0.37	2.45E-04	0.15	2.36E-01	MHC class I antigen processing & presentation
CALR	0.16	1.48E-01	0.21	5.20E-02	0.00	9.87E-01	0.02	8.64E-01	-0.01	9.31E-01	-0.01	9.65E-01	MHC class I antigen processing & presentation
PSMB8	0.37	6.03E-04	0.51	2.17E-05	0.40	1.89E-02	0.45	1.43E-04	0.24	9.80E-02	0.49	1.36E-02	MHC class I antigen processing & presentation
PSMB9	0.64	9.17E-03	0.75	9.52E-04	0.63	4.60E-02	0.48	5.47E-04	0.33	4.96E-02	0.65	4.46E-03	MHC class I antigen processing & presentation
PSMB10	0.31	3.26E-04	0.32	4.57E-04	0.35	5.70E-03	0.28	1.61E-02	0.27	2.13E-02	0.61	8.59E-06	MHC class I antigen processing & presentation
PSME1	0.41	5.47E-08	0.37	4.04E-06	0.30	1.84E-03	0.40	3.50E-08	0.40	4.13E-06	0.41	5.03E-05	MHC class I antigen processing & presentation
PSME2	0.36	4.50E-06	0.33	2.25E-04	0.29	1.68E-02	0.38	9.25E-07	0.54	5.63E-10	0.45	7.71E-06	MHC class I antigen processing & presentation
HLA-A	0.18	1.01E-01	-0.02	9.04E-01	0.09	6.64E-01	0.09	3.16E-01	-0.01	9.38E-01	0.13	3.98E-01	MHC class I antigen processing & presentation
HLA-B	0.14	1.59E-01	-0.02	8.76E-01	0.04	8.57E-01	0.12	2.11E-01	0.00	9.95E-01	0.24	1.98E-01	MHC class I antigen processing & presentation
HLA-C	0.01	9.61E-01	-0.34	1.21E-01	0.13	7.49E-01	0.09	2.90E-01	-0.07	5.82E-01	0.20	2.59E-01	MHC class I antigen processing & presentation
ERAP1	-0.17	4.77E-01	-0.33	1.83E-01	0.22	4.22E-01	0.11	6.38E-01	0.05	8.24E-01	-0.09	6.79E-01	MHC class I antigen processing & presentation
ERAP2	0.00	9.91E-01	-0.10	7.69E-01	0.41	2.77E-01	0.36	1.73E-01	0.09	7.86E-01	-0.18	4.87E-01	MHC class I antigen processing & presentation
NLRC5	0.25	1.35E-01	0.02	9.41E-01	-0.08	7.61E-01	n.a.	n.a.	n.a.	n.a.	n.a.	n.a.	MHC class I antigen processing & presentation
IRF2	0.01	9.44E-01	0.02	8.84E-01	0.00	9.79E-01	0.16	5.70E-02	0.01	9.60E-01	0.10	2.95E-01	MHC class I antigen processing & presentation
STAT1	0.30	8.17E-03	0.00	9.82E-01	0.23	1.87E-01	0.18	1.27E-01	0.05	7.58E-01	0.13	4.53E-01	MHC class I antigen processing & presentation
HLA-DOB	0.85	1.03E-03	0.73	3.54E-04	0.53	4.47E-02	0.26	4.44E-02	0.38	5.92E-04	0.18	7.95E-02	MHC class II antigen presentation
HLA-DMA	0.44	5.28E-03	0.21	2.07E-01	0.15	5.62E-01	0.48	3.62E-04	0.40	1.70E-02	0.71	4.08E-03	MHC class II antigen presentation
HLA-DMB	0.27	1.51E-02	0.20	2.06E-01	-0.02	9.44E-01	0.30	1.75E-02	0.23	1.64E-01	0.19	3.83E-01	MHC class II antigen presentation
ENTPD1	-0.79	1.41E-05	-0.43	1.83E-02	-0.59	5.10E-03	-0.32	1.44E-04	-0.50	1.47E-09	-0.24	1.38E-02	adenosine-mediated immune suppression
NT5E	-1.48	2.25E-10	-1.65	6.74E-11	-1.00	4.79E-04	-1.16	1.43E-13	-1.30	5.48E-11	-0.65	1.79E-03	adenosine-mediated immune suppression
IL10	-0.43	2.07E-03	-0.39	5.22E-03	-0.39	5.10E-03	-0.04	5.29E-01	-0.17	1.36E-05	-0.05	3.31E-01	IL10-mediated immune suppression
TGFB1	-0.81	9.27E-07	-0.86	3.32E-07	-0.59	7.81E-03	-0.26	8.91E-03	-0.52	7.24E-08	-0.22	4.30E-02	TGF-beta pathway activation/fibroblast activation
TGFB3	-1.48	3.46E-07	-1.64	8.14E-10	-1.13	4.82E-06	-0.60	4.94E-14	-0.43	7.81E-08	-0.49	6.78E-07	TGF-beta pathway activation
WNT4	-1.28	3.50E-08	-1.08	3.98E-07	-1.12	9.38E-06	-0.28	3.70E-04	-0.33	1.48E-06	-0.45	2.07E-05	Wnt pathway activation
WNT7A	-1.57	1.09E-05	-1.53	7.61E-05	-0.25	6.93E-01	-0.41	3.80E-03	-0.23	2.28E-01	0.06	7.71E-01	Wnt pathway activation/fibroblast activation
FAP	-2.13	7.26E-11	-2.59	4.27E-13	-1.45	5.68E-05	-1.34	2.11E-10	-1.49	8.76E-10	-0.49	3.72E-02	cancer-associated fibroblasts
PDGFRB	-1.39	1.58E-15	-1.30	3.26E-11	-0.89	9.38E-07	-0.62	2.46E-11	-0.65	4.08E-09	-0.57	3.07E-07	cancer-associated fibroblasts
PDGFRA	-2.12	1.03E-11	-2.02	8.50E-11	-1.27	5.77E-05	-1.39	2.29E-12	-1.64	2.92E-12	-1.31	1.82E-07	cancer-associated fibroblasts
INHBA	-1.77	4.91E-12	-2.16	4.30E-12	-1.29	5.99E-05	-1.21	2.10E-08	-1.52	6.69E-10	-0.65	3.71E-03	immunotherapy resistance/fibroblast activation
CD274	0.09	7.22E-01	-0.06	8.05E-01	0.32	1.08E-01	n.a.	n.a.	n.a.	n.a.	n.a.	n.a.	Immune Checkpoint
